# Partitioning of the denitrification pathway and other nitrite metabolisms within global oxygen deficient zones

**DOI:** 10.1038/s43705-023-00284-y

**Published:** 2023-07-20

**Authors:** Irene H. Zhang, Xin Sun, Amal Jayakumar, Samantha G. Fortin, Bess B. Ward, Andrew R. Babbin

**Affiliations:** 1 Department of Earth, Atmospheric and Planetary Sciences, Massachusetts Institute of Technology, Cambridge, MA USA; 2 Program in Microbiology, Massachusetts Institute of Technology, Cambridge, MA USA; 3 Department of Global Ecology, Carnegie Institution for Science, Stanford, CA USA; 4 Department of Geosciences, Princeton University, Princeton, NJ USA

**Keywords:** Biogeochemistry, Microbial ecology

## Abstract

Oxygen deficient zones (ODZs) account for about 30% of total oceanic fixed nitrogen loss via processes including denitrification, a microbially mediated pathway proceeding stepwise from NO_3_^–^ to N_2_. This process may be performed entirely by complete denitrifiers capable of all four enzymatic steps, but many organisms possess only partial denitrification pathways, either producing or consuming key intermediates such as the greenhouse gas N_2_O. Metagenomics and marker gene surveys have revealed a diversity of denitrification genes within ODZs, but whether these genes co-occur within complete or partial denitrifiers and the identities of denitrifying taxa remain open questions. We assemble genomes from metagenomes spanning the ETNP and Arabian Sea, and map these metagenome-assembled genomes (MAGs) to 56 metagenomes from all three major ODZs to reveal the predominance of partial denitrifiers, particularly single-step denitrifiers. We find niche differentiation among nitrogen-cycling organisms, with communities performing each nitrogen transformation distinct in taxonomic identity and motility traits. Our collection of 962 MAGs presents the largest collection of pelagic ODZ microorganisms and reveals a clearer picture of the nitrogen cycling community within this environment.

## Introduction

Bioavailable (i.e., fixed) nitrogen limits biological productivity in much of the surface ocean, thus the processes that balance the nitrogen budget require a thorough understanding [1]. Microorganisms mediate the biogeochemical transformations of nitrogen in its various forms, including the removal of fixed nitrogen via denitrification and anaerobic ammonium oxidation (anammox). Both of these processes occur when oxygen is limiting, and thus global oxygen deficient zones account for an estimated fixed nitrogen loss of 50–77 Tg N yr^−1^, about 30% of the oceanic total, despite containing only 0.1–0.2% of oceanic volume [2]. The three major oceanic oxygen deficient zones (ODZs) are located in the eastern tropical North Pacific (ETNP), the eastern tropical South Pacific (ETSP), and the Arabian Sea. Although denitrification and anammox both occur within all 3 ODZs, investigations into the rates and relative contributions of each process indicate anammox frequently predominates in the ETSP and ETNP [3–5], while denitrification exceeds anammox in the Arabian Sea [5, 6].

**Fig. 1:**
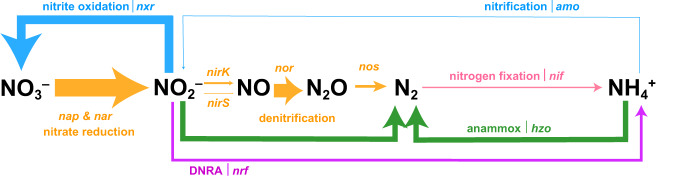
Schematic of nitrogen cycling within ODZ depths (O_2_ < 3 μM). Thickness of arrows correlates to total relative abundance of MAGs with the gene encoding the enzyme for that pathway, averaged across ODZ depths in the ETSP, ETNP, and Arabian Sea. Genes queried as proxies for each metabolism are italicized next to the name of the metabolism. Figure modified from Babbin et al. 2021 [122].

Denitrification is typically a stepwise process of anaerobic respiration in heterotrophs that can use nitrogen oxides as terminal electron acceptors through enzymes encoded by a suite of known genes (Fig. 1). The first step of nitrate (NO_3_^–^) reduction to nitrite (NO_2_^–^) occurs via the periplasmic NO_3_^–^ reductase encoded by *nap* or the membrane-bound NO_3_^–^ reductase encoded by *nar*. The second step, NO_2_^–^ reduction to nitric oxide (NO), is catalyzed by two functionally similar but structurally distinct NO_2_^–^ reductases, a copper-containing NO_2_^–^ reductase and a cytochrome *cd*_1_-containing NO_2_^–^ reductase encoded by *nirK* and *nirS*, respectively. The reduction of NO to nitrous oxide (N_2_O) proceeds through NO reductases encoded by diverse *nor* genes [7–9], and N_2_O is finally reduced to inert N_2_ gas by N_2_O reductases encoded by *nos* [10]. NO may also be disproportionated to N_2_ and O_2_ by the putative nitric oxide dismutase gene encoded by *nod* in a process of oxygenic denitrification [11–13]. Denitrification has been demonstrated to be a modular process, with some microorganisms able to perform the complete reduction of NO_3_^–^ to N_2_ while others are only capable of one or a subset of steps [9, 14, 15]. Increasingly, sequencing efforts demonstrate that diverse partial denitrifiers comprise the majority of environmental denitrifiers, yet denitrification is often treated as a complete, singular process in marine biogeochemistry [2, 16–19].

Denitrification intermediates interface with various other nitrogen metabolisms; for example, NO_2_^–^ participates in pathways for dissimilatory nitrate reduction to ammonia (DNRA), NO_2_^–^ oxidation to NO_3_^–^, and anammox (Fig. 1). As partial denitrifiers either produce or consume key denitrification intermediates depending on their specific genetic makeup, they may hold particular relevance for the marine nitrogen cycle. Furthermore, the emission of N_2_O, a major greenhouse gas, has been correlated with the presence of partial denitrifiers lacking *nos*, while N_2_O consumption might correlate with the presence of *nos*-carrying partial denitrifiers [15, 20]. ODZs are a major source of N_2_O, yet the magnitude and net effect of potential N_2_O emission and consumption within ODZs remains debated [21–24]. An understanding of the linkages within the marine nitrogen cycle thus requires determining the genetics, metabolisms, and identities of partial denitrifiers within ODZs.

Previous estimates of the marine nitrogen budget indicate an excess of fixed nitrogen losses over gains [25–27], although more recent models accounting for spatial variability of nitrogen loss processes within suboxic zones imply a balanced budget [28]. Important uncertainties remain in the relative contributions of nitrogen loss processes, nitrogen fixation, and fixed nitrogen recycling via nitrification, NO_2_^–^ oxidation, and DNRA. As each of these processes operates upon a specific set of nitrogen and carbon substrates, their spatial variability and coupling within and across ODZs may depend upon the magnitude and stoichiometry of these substrates [29, 30]. Sequencing [9, 31, 32] and modeling [33] reveal a potentially large contribution of N_2_O and N_2_ by denitrifiers on particles, which are characterized by distinct organic matter stoichiometries and oxygen gradients compared to the water column. Since particle colonization is linked to motility and chemotaxis capabilities in marine microorganisms [34, 35], this suggests that chemotaxis and motility traits might be also widespread in denitrifiers.

Metagenome sequencing efforts have revealed a diversity in denitrification genes missed by previous PCR primer-based studies [36, 37], including divergent denitrification genes and taxa within ODZs [9, 15, 38]. Prevalent horizontal transfer of denitrification genes precludes robust taxonomic assignments of denitrifiers from metagenomes, as well as determination of which genes co-occur within the same organism [14, 39, 40]. Metagenome-assembled genomes (MAGs) from ODZs have uncovered nitrogen cycling capabilities within various understudied or uncultivated taxa [38, 41–44], including diverse partial denitrifiers [45]. However, these studies focus on a limited number of taxa and MAGs, and few MAGs have been assembled from the Arabian Sea ODZ. We assemble the largest collection of uncultured microbial genomes from the ETNP and Arabian Sea ODZs to date. By assessing these MAGs for denitrification and other nitrogen cycling genes and comparing their relative abundances across all three global pelagic ODZs, we reveal the taxonomic identity of ODZ denitrifiers, their nitrogen cycling capabilities within the water column, and their potential impacts on biogeochemistry.

## Materials and methods

### Sampling, sequencing, and read quality

**Fig. 2:**
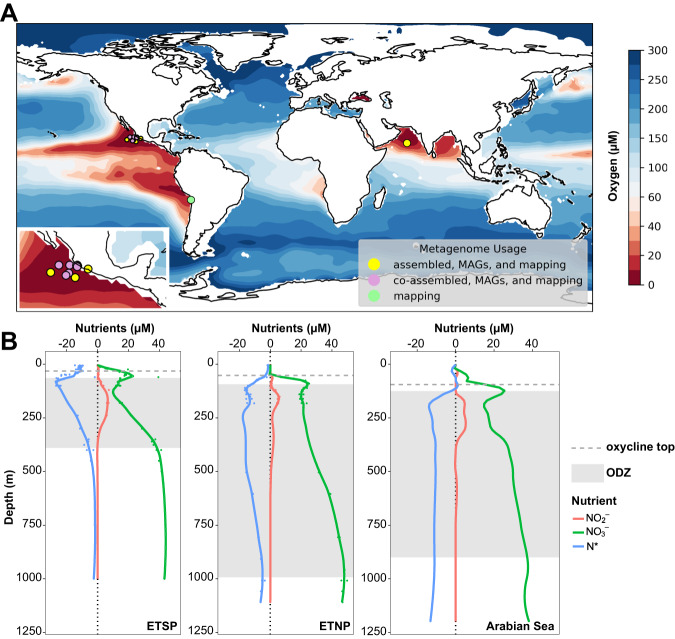
Biogeochemical context of oxygen deficient zone samples. **A** Locations of metagenomes from ETNP, ETSP, and Arabian Sea. Colors correspond to the concentration of dissolved oxygen at 300 m below sea surface according to World Ocean Atlas 2018. **B** Representative profiles for NO_3_^–^, NO_2_^–^, and N* for all 3 major ODZs. ETSP [31] and ETNP [9] profiles are composite profiles from multiple sampling sites for published metagenomes, while Arabian Sea profile [6] is from sampling station 2 corresponding to Arabian Sea metagenomes used in this study. Dotted gray horizontal line indicates the top of the oxycline at each station based upon corresponding O_2_ CTD data, and shaded gray box indicates the ODZ extent (O_2_ < 3 μM). A full set of O_2_ profiles can be found in Supplementary Fig. S1.

Metagenomic analysis was performed for 11 stations in the three major oceanic ODZs. Sampling locations for each metagenome were visualized using Python 3.7.12 and the cartopy package. These were plotted against global oxygen concentrations from 300 m below sea surface from Ocean Data Atlas 2018 (Fig. 2A). Data for vertical profiles of O_2_, NO_3_^–^, and NO_2_^–^ were retrieved from the original studies when available or from BCO-DMO (Fig. 2B, S1). From NO_3_^–^, NO_2_^–^, and PO_4_^3–^ data, N* was calculated using the equation N*= (NO_3_^–^ + NO_2_^–^ – 16 × PO_4_^3–^) + 2.9 µmol kg^−1^ [46]. Oxygen measurements were made using a rosette containing a Conductivity-Temperature-Depth (CTD) profiler equipped with a Seabird Clark-type dissolved oxygen electrode with a resolution in the micromolar oxygen range. While previous measurements with high resolution trace oxygen sensors show oxygen below the detection limit of 10 nM in ODZ cores [47, 48], the gradients exhibited by Clark-type electrodes remain valid for identifying ODZ waters [49]. As the minimum oxygen concentrations reported by CTD electrodes range from 0–2.9 μM between datasets (Supplementary Dataset S1), we define ODZ depths as those with oxygen <3 μM. Vertical profiles for O_2_, NO_3_^–^, NO_2_^–^, and N* were graphed using R ggplot2 (Fig. 2B, S1). Due to sparsity of NO_3_^–^ and NO_2_^–^ data for individual stations in the ETNP and ETSP, profiles were aggregated to create a composite profile for each study, and smoothed using geom_smooth with the LOESS method in R ggplot2.

For 2016 ETNP, 2018 ETNP, and 2007 Arabian Sea metagenomes reported here for the first time, samples were collected with Niskin bottles and filtered onto 0.22 μm Sterivex filters with peristaltic pumps. Volumes of seawater filtered are as follows: 3.5–4.5 L (2016 ETNP), 2.8–7 L (2018 ETNP), and 4.7–5.5 L (2007 Arabian Sea). Filters were subsequently frozen in liquid nitrogen, dry ice, or at −80 °C until extraction. DNA extractions were performed with the Qiagen AllPrep DNA/RNA Mini Kit following manufacturer protocols. Sequencing was performed at the DOE Joint Genome Institute (JGI) on a NovaSeq (Illumina) resulting in paired end reads 151 base pairs in length. BBDuk v38.45 was used to remove contaminants, trim adapters, and remove low quality bases. Reads mapping to human and animal references as well as common microbial contaminants were removed using the JGI pipeline, and final reads were retrieved from JGI.

Sampling and sequencing methods for public ETNP [9, 43, 50] and ETSP [31, 51] metagenomes are described elsewhere. Raw samples were downloaded from the Sequence Read Archive (SRA) using NCBI BioProject ID numbers PRJNA350692, PRJNA254808, PRJNA323946, PRJNA68419, and PRJNA217777 (Table S1). For all metagenomes, only metagenomes from >0.22 μm and 0.22–1.6 μm size fractions were retained, while metagenomes from size fractionated samples or particle-associated fractions were removed from further analysis. Raw reads were trimmed with Trimmomatic v0.39 to remove adapters and low-quality bases using the LEADING:3 TRAILING:3 SLIDINGWINDOW:4:15 MINLEN:36 flags [52].

### Metagenome assembly and binning

Reads were assembled with MEGAHIT v1.2.9 [53] using default parameters. For metagenomes first described here from 2016 ETNP, 2018 ETNP, and 2007 Arabian Sea collections, each metagenome was assembled separately. For public ETNP metagenomes, all samples from the same study were co-assembled to maximize read depth (Supplementary Dataset S2). Low sequencing depths for ETSP metagenomes prevented recovery of high-quality assemblies and bins, but reads from ETSP metagenomes were retained for mapping to our final set of MAGs.

From each ETNP and Arabian Sea assembly, contigs shorter than 800 nucleotides were removed, Bowtie v2.3.5 was used to map reads to assemblies, and samtools v1.7 sorted and indexed the resultant bam files. Binning was performed using CONCOCT v1.00 [54], Metabat2 v2.12.1 [55], and Maxbin2 v2.2.6 [56] within the metaWRAP v1.3 wrapper [57]. From the three bin sets per assembly, the best non-redundant bins were selected using the metaWRAP bin_refinement module. Resulting bins were further improved with the metaWRAP reassemble_bins module, in which reads belonging to each bin are extracted and reassembled with both a “strict” and “permissive” algorithm, and only bins which are improved by this reassembly are altered in the final set of bins. Bin quality was assessed using CheckM v1.0.12 and metagenome-assembled genomes (MAGs) were defined as bins with completion >50% and contamination <10% [58].

### Gene searching within metagenomes

For each metagenome, assemblies were annotated with PROKKA v1.14.6 against the HAMAP [59] and Pfam databases [60]. Annotated assemblies were queried with Hidden Markov Models (HMMs) using HMMer3 [61] for the denitrification genes *napA, narG, nirK, nirS*, and *nosZ*, as well as the DNRA gene *nrfA*, the nitrogen fixation gene *nifH*, the NO_2_^–^ oxidation gene *nxrA*, the key gene for ammonia oxidation *amoA* for ammonium-oxidizing archaea and bacteria separately, the NO dismutase gene *nod*, and the anammox gene *hzo*. For *napA, narG, nirK, nirS*, and *nosZ* HMMs, protein sequences were obtained from the NCBI Conserved Domain Database (CDD) containing well-annotated, curated protein families, and from UniRef90 [62]. Protein sequences were aligned with MAFFT v7.450 with the --linsi parameter [63] and manually checked for alignment quality. HMMs were built using *hmmbuild* implemented in HMMer3 [61]. HMMs for *hzo* and *nxrA* were obtained from a published collection of metabolic HMMs [64], while HMMs for *nifH, nrfA*, and *amoA* were obtained from the FunGene repository [65]. For NO reductases, individual HMMs for canonical *qnor* and *cnor* variants, along with HMMs for non-canonical *bnor, enor, gnor, nnor*, and *snor* genes and the NO dismutase gene *nod*, were downloaded from a previous publication [7, 66]. All HMMs were validated against protein sequences for respective proteins downloaded from the NCBI RefSeq non-redundant protein collection.

As *narG* and *nxrA* share high sequence similarity due to their complex evolutionary history [67], we performed an additional phylogenetic analysis to validate putative *nxrA* sequences. Known *nxrA*-encoded proteins from phylogenetically diverse nitrite oxidizers and anammox bacteria were obtained from the NCBI database, along with related proteins for perchlorate reductase, arsenate reductase, formate reductase, periplasmic nitrate reductase encoded by *napA*, and respiratory nitrate reductase encoded by *narG* [68]. These were aligned with putative ODZ *nxrA* sequences using MAFFT v7.450 with the—linsi parameter [63]. Alignments were trimmed with trimAl v1.4.rev15 using the -automated1 flag [69]. IQTree v.1.6.12 was used to reconstruct maximum likelihood phylogenetic trees [70] using the LG + R8 model with 1000 ultrafast bootstraps [71]. Branch supports were calculated with the SH-like approximate likelihood ratio test (SH-aLRT) with 1000 bootstraps. We identified *nxrA* proteins as those whose nearest relatives within a clade were identified as *nxrA* (Fig. S2).

Metagenome assemblies were searched using HMMs for the single-copy genes (SCGs) *rpoB, rplB, gyrB, rpS3*, and *recA*, which are conserved in bacteria and archaea. HMMs for *rplB, gyrB*, and *recA* were retrieved from the FunGene repository [65], and the *rpS3* HMM from a published study [72]. The *rpoB* HMM was created following above methods using *rpoB*-encoded protein sequences from CDD and UniRef90. Normalization was performed by dividing the numbers of hits for each nitrogen cycling gene by the average number of hits for the five SCGs per metagenome assembly. As relatively small differences are reported for average genome sizes across the depths our metagenomes span [73], we do not expect genome size variations to significantly affect our results. Pearson correlations were done for all normalized percentages of nitrogen cycling genes with each other and with O_2_, NO_3_^–^, NO_2_^–^, depth, and rate measurements for relevant nitrogen cycling reactions from the same sampling sites. The corr.test function in the R psych package v2.2.9 was used to calculate the Pearson correlations and test statistics (*p*-value) using a two-tailed t-test to determine if correlations differ significantly from 0. We corrected for multiple hypothesis testing with the Benjamini–Hochberg method [74], and a significance threshold of *p* < 0.01 was chosen. Correlation heatmaps were visualized with the R heatmaply package 1.4.0.

### MAG analyses, mapping, and gene searching

Taxonomy was assigned to all MAGs using GTDB-tk v1.7.0 [75] with the classify_wf workflow. MAGs were annotated with PROKKA v1.14.6 [76] against the HAMAP [59] and Pfam databases [60]. For coverage mapping and comparison purposes, MAGs were dereplicated with dRep v3.2.2 with the -sa 0.99 flag to produce a final collection of non-redundant genomes. Coverage mapping was performed with CoverM on dereplicated genomes using the flags minimap2-sr --min-read-aligned-percent 50 --min-read-percent-identity 0.95 --min-covered-fraction 0 (https://github.com/wwood/CoverM). Relative abundances of dereplicated MAGs resulting from CoverM mapping were visualized using R v4.1.3 and the packages phyloseq, ggplot2, and dplyr (Fig. S3).

We minimized the risk of protein misidentification (false positives) while maximizing the retrieval of non-canonical genes or more distant homologues using HMMs along with examination of gene hits for the presence of conserved active site residues. PROKKA annotations for each MAG were queried for nitrogen cycling genes using curated HMMs as described above. From positive hits for each gene, the corresponding protein sequences were retrieved and multiple sequence alignments generated with MAFFT v7.450 using the --auto and --leavegappyregion parameters. These alignments were visualized in JalView v2.11.2.6 [77] and inspected for alignment quality and the conservation of active site amino acid residues for each protein. These active sites are: the *napA* [4Fe-4S] cluster and molybdenum active site [78], the *narG* N-terminal cysteine consensus motif [79], the *nirK* type I and type II copper centers[37, 80], the *nirS* cytochrome d_1_ heme binding site [37], the *nosZ* Cu_A_ and Cu_Z_ copper binding sites [81], and the heme and Fe_B_ active sites and protein channels in various *nors* [7, 82]. Validated hits were used to generate diagrams of gene presence and absence for each MAG. The number of MAGs belonging to each nitrogen cycle gene combination was visualized using R v4.1.3 and the UpSetR package. Additionally, relative abundances of MAGs carrying each gene across all metagenomes were visualized with the R packages phyloseq, ggplot2, and dplyr. Euler diagrams showing relative abundances of MAGs carrying each denitrification step for each metagenome were calculated by adding up relative abundances of all MAGs capable of each of the four denitrification steps, and visualized with the R eulerr package.

For bacterial motility, chemotaxis, and aerotaxis genes, HMMs were downloaded from a previous publication [83]. The chemotaxis genes *cheA*, *cheB*, and *cheR*, the aerotaxis receptor gene *aer*, and the genes *fliG, fliM*, and *fliNY* involved in bacterial flagellar motion were searched against all MAGs. In contrast to nitrogen cycling genes, the chemotactic and motility apparatus in bacteria and archaea comprises a number of interacting components and genes, not all of which may be required for a given organism to be motile or chemotactic [83]. To account for this and varying MAG completeness, chemotaxis or motility were considered present if at least 2 out of the 3 queried core chemotaxis or motility genes were present in the MAG. Additionally, sequences for the archaeal flagellar gene *flaJ* were downloaded from the NCBI conserved domain database (CDD) and an HMM for *flaJ* was built following methods above. We used this HMM to search against all MAGs, and archaea possessing this gene were considered motile.

## Results

### Biogeochemical context and patterns of metagenome gene abundances

Oxygen minimum zones (OMZs) are common subsurface features in the ocean, and even strong minima can still maintain several micromolar oxygen and support aerobic processes. We define an oxygen depleted zone (ODZ) as a region within the OMZ characterized by oxygen depletion below levels detectable with conventional electrodes or Winkler titrations, and typically signified by the presence of a secondary nitrite maximum. For all 3 ODZ regions, oxygen profiles from each sampling station show a characteristic rapid decrease from surface saturation to anoxia, termed the oxycline, occurring approximately between 50–100 m depth (Fig. 2B, S1). Below the oxycline, oxygen levels remain beneath the detection limit until around 500 m below the sea surface for the ETSP and 1000 m below the sea surface for the ETNP and Arabian Sea. Corresponding profiles of NO_3_^–^ and NO_2_^–^ reveal typical decreases in NO_3_^–^ and increases in NO_2_^–^ (the secondary nitrite maximum, or SNM) in this region (Fig. 2B). N* values become increasingly negative within the oxycline and ODZ, indicating a net nitrogen deficit relative to phosphorus characteristic of the nitrogen loss processes denitrification and anammox. The ETSP ODZ is the narrowest of the 3 ODZs, but displays the strongest N* deficit indicating intense nitrogen loss processes. The ETNP and Arabian Sea, in contrast, display thick water column ODZs yet have comparatively less intense N* deficits (Fig. 2B). The secondary nitrite maximum and NO_3_^–^ consumption are greatest in the ETSP and least intense in the ETNP.

**Fig. 3:**
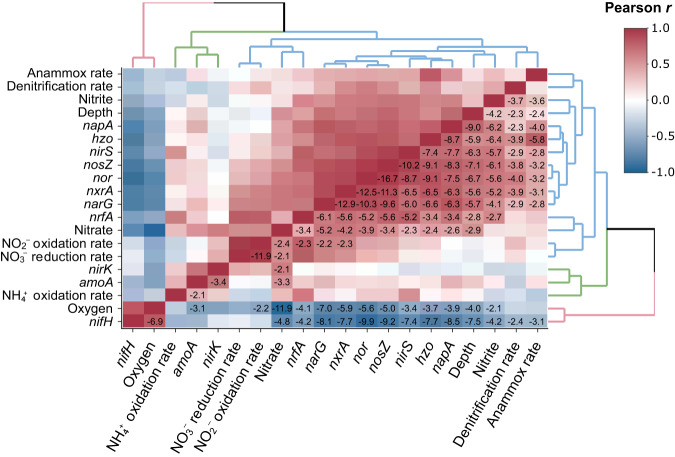
Statistical relationships among metadata and gene abundances. Pearson correlation coefficients for all relevant nitrogen cycling genes, rates, and concentrations of nitrate, nitrite, and oxygen with log_10_-transformed *p*-values overlaid, showing only significant *p*-values (*p* < 0.01) after a Benjamini–Hochberg multiple hypothesis correction.

The distribution of denitrifying genes and MAGs was investigated in 56 metagenomes spanning multiple depths, years, sites, and studies from the three permanent, open ocean ODZs (Fig. 2A). Within the mixed, oxygenated layers above the ODZ, denitrification genes in the individually assembled metagenomic contigs are scarce, but increase in abundance at the deeper hypoxic and anoxic waters of the oxycline and ODZ (Fig. S4A). While the *nirK* gene, encoding copper-containing NO_2_^–^ reductase, peaks in abundance at 100 m below sea surface, the *nirS* cytochrome *cd*_1_-containing NO_2_^–^ reductase*, nor* NO reductase, and *nosZ* N_2_O reductase genes peak below 150 m. Pearson correlations reveal a significant positive correlation among these three genes (*p* < 0.01), along with the NO_3_^–^ reductase genes *napA* and *narG*, the anammox hydrazine oxidoreductase gene *hzo*, the NO_2_^–^ oxidation gene *nxrA*, and the DNRA gene *nrfA*. These genes, along with nitrate and nitrite concentration, also exhibit significant positive corrections with rates of denitrification and anammox, and the strongest correlation with anammox rate is *hzo* (*p* = 1 × 10^–6^). In contrast, *nirK* exhibits little correlation with other denitrification genes, yet correlates positively with the *amoA* gene for ammonia monooxygenase (*p* = 0.0004) (Fig. 3). The majority of *nirK* reads around 100 m belong to the archaeal *nirK* type associated with Thaumarchaeota [84], thus driving this correlation of *amoA* and *nirK*. Nitrate concentration is significantly correlated with oxygen, *nirK*, *amoA*, and the rates of NO_2_^–^ oxidation and NO_3_^–^ reduction, among others.

### ODZ MAG collection and denitrifying community stability

To determine if these positive correlations between *napA, narG, nirS, nor*, and *nosZ* are due to co-occurring organisms or co-occurrence of these genes within the same organisms, we generated 962 MAGs >50% completion and <10% contamination from the ETNP and Arabian Sea, with 245 MAGs >90% completion. The majority of our MAGs (814) are under 5% contamination, with an average contamination of 2.4% across all MAGs. After dereplication, these MAGs contain 307 uncharacterized taxa at the species level out of 497 non-redundant genomes, including 1 MAG novel at the phylum level, 2 at the class level, 8 at the order level, and 19 at the family level (Supplementary Dataset S3), as determined by the lowest level taxonomic assignment performed by GTDB-tk. Mapping non-redundant MAGs from our collection to all three global ODZs reveals a distinctive and diverse ODZ community that is stable at the phylum level across time, sampling sites, and ODZs (Fig. S3). Within ODZ depths, up to approximately 50% of assembled reads map to MAGs, while mapping rates are lower above and below the ODZ. In anoxic layers, *Proteobacteria* (*Alphaproteobacteria* and *Gammproteobacteria*) are the most abundant, followed by *Marinisomatota*. Phyla present in lower abundance across most or all metagenomes include *Actinobacteria, Planctomycetes, SAR324, Nitrospinota*, and *Thermoplasmatota* (Fig. S3).

**Fig. 4:**
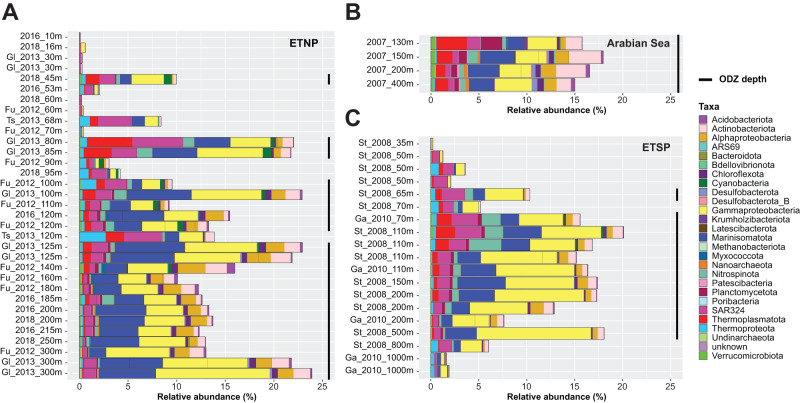
Relative abundances of denitrifier MAGs across metagenomes. Relative abundances are color-coded by phylum-level taxonomy, except for *Proteobacteria,* which is colored by class, from the three major ODZs: (**A**) Eastern Tropical North Pacific, (**B**) Arabian Sea, and (**C**) Eastern Tropical South Pacific. Black bars at the right of the panels indicate ODZ depths (O_2_ < 3 μM).

Mapping dereplicated ETNP and Arabian Sea MAGs to metagenomes from all three ODZs reveals a similar pattern of phylum-level abundance, distribution, and stability in denitrifying communities, defined as MAGs harboring at least one denitrification gene, across the ETSP and ETNP (Fig. 4). Arabian Sea metagenomes also reveal similar proportions as the ETNP and ETSP at similar depths, but the limited number of sampling depths restricts comparison. Denitrifier draft genomes represent up to 24% of the ODZ microbial community by relative abundance, and likely more as 50% or more of the community did not map to our MAG collection. *Proteobacteria* and *Marinisomatota* dominate within denitrifiers, but the denitrifying community includes 22 of the 34 total phyla into which all MAGs were assigned (Fig. 4).

### Denitrifying phyla and relative abundances within the ODZ water column

**Fig. 5:**
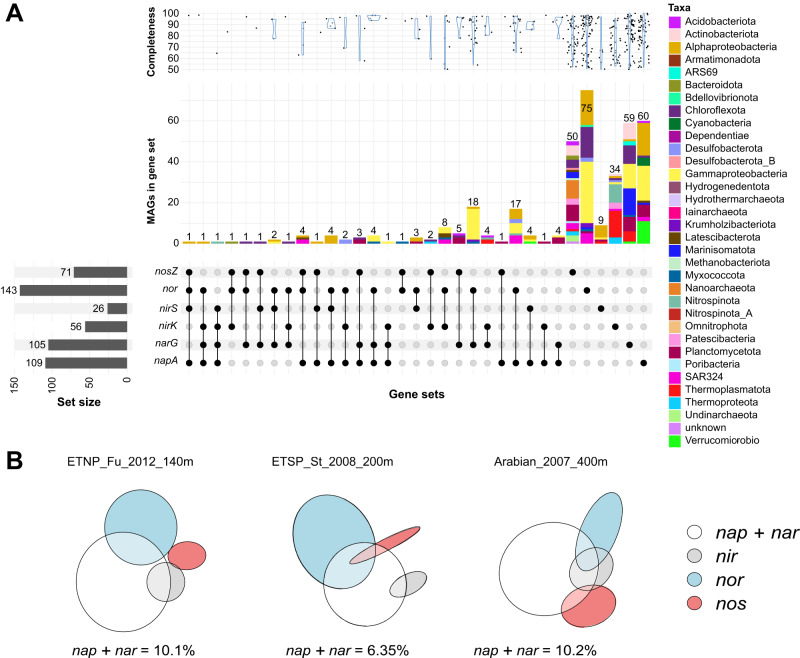
Denitrification gene composition of ODZ denitrifiers. **A** The number of MAGs carrying each denitrification gene set within the ODZ MAG collection. Top panel shows genome completion distributions for MAGs belonging to each gene set, middle panel shows the number of MAGs colored by phylum-level taxonomy, with the exception of *Proteobacteria* which is colored by class. Bottom panel shows the genes within each gene set. Left bottom panel shows the number of MAGs carrying each specific nitrogen cycle gene. Additional graphs showing all nitrogen cycling genes can be found in Supplementary Fig. S5B. **B** Representative Euler diagrams for ODZ depths showing the relative abundance and co-occurrence of the four steps of denitrification. Circles and intersections are scaled to the total relative abundance of all MAGs possessing the genes for that step or step combination. The white circle corresponds to the relative abundance of MAGs with *napA, narG*, or both within that metagenome. All Euler diagrams for all metagenomes can be found in Supplementary Fig. S10.

**Fig. 6:**
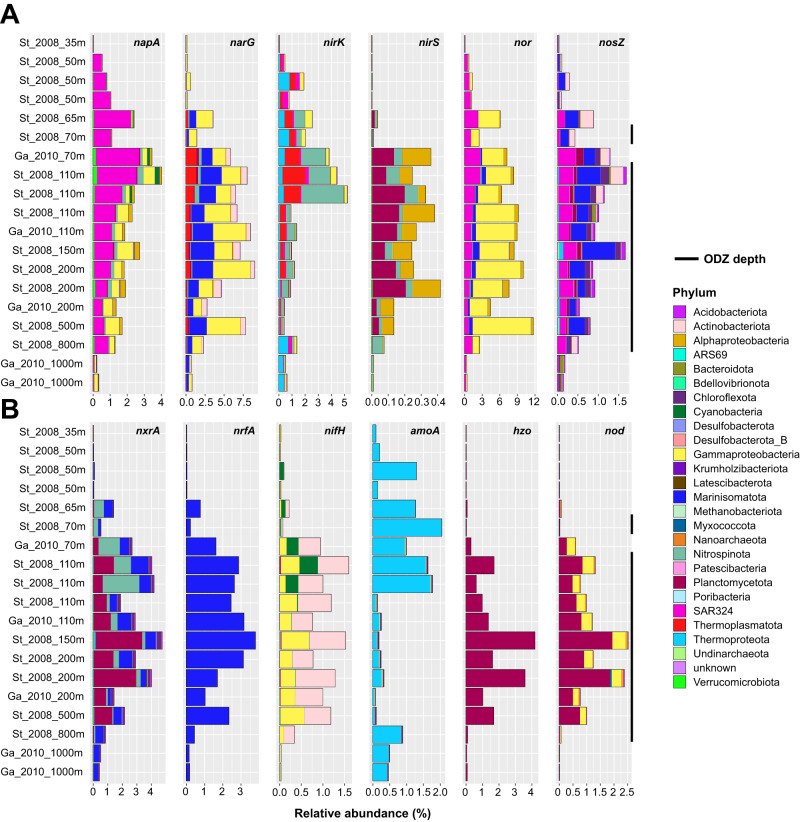
Relative abundance mapping of MAGs for nitrogen metabolisms in the ETSP. **A** Relative abundance mapping of MAGs carrying each denitrification gene across all ETSP metagenomes. MAGs are colored by phylum-level taxonomy, except for *Proteobacteria,* which is colored by class. **B** Relative abundance mapping of MAGs carrying each non-denitrification nitrogen cycling gene across all ETSP metagenomes. Black bars to the right of the graphs indicate ODZ depths (O_2_ < 3 μM). Additional graphs for ETNP and Arabian Sea metagenomes can be found in Supplementary Figs. S6 and S7, respectively.

Single-step *napA* or *narG* NO_3_^–^ reducers, single-step *nor* NO reducers, and single-step *nosZ* N_2_O reducers dominate in number of MAGs. 105 ODZ MAGs (11%) have *narG*, with 59 (58% of narG MAGs) possessing no other denitrification genes (Fig. 5A). Similarly, *napA* MAGs account for 109 MAGs total, and 60 (55%) of these have only *napA*. The overlap of *napA* and *narG* in MAGs is rare (14 MAGs). A total of 200 (21%) MAGs have NO_3_^–^ reduction capability, and the majority of both types of NO_3_^–^ reducers possess no further denitrification capabilities (Fig. 5). In terms of relative abundance, *napA* NO_3_^–^ reducers reach about 5% of the community in the ODZ core, while *narG* NO_3_^–^ reducers reach about 12%, (Fig. 6A and Supplementary Figs. S6A, S7). While *narG* nitrate reducers coexist with *napA* NO_3_^–^ reducers across depths, *Proteobacteria* and *SAR324* dominate within *napA* NO_3_^–^ reducers, while *Marinisomatota* and *Proteobacteria* comprise the most abundant fractions of *narG* NO_3_^–^ reducers. Within our collection, 10 Marinisomatota MAGs from the ETNP and 3 from the Arabian Sea belonging to diverse families (GTDB taxonomy D37C17, UBA2128, UBA1611, TCS55, and S15-B10) harbored *narG*. Within metagenome assemblies, *narG* comprises up to 40% of gene hits normalized to a set of single-copy housekeeping genes (SCGs) (Fig. S4A), the most abundant gene within our queried set. The *napA* gene is the second most commonly found denitrification gene within the metagenomes at around 30% abundance compared to our SCGs, while *nirS* and *nirK* comprise 10% or less at ODZ depths (Fig. S4A).

Consistent with the relatively high abundance of *nor* in metagenomes (up to 25% at ODZ depths), 143 MAGs carried a variety of *nor*, with 75 (53%) possessing no other denitrification genes (Fig. 5A). However, of these only 62 carry the canonical variants *qnor* and *cnor*, while 91 carry a non-canonical *nor* (Fig. S8A), and MAGs carrying non-canonical *nor* reach higher relative abundance in the ETNP and ETSP (Fig. S8). *Proteobacteria, SAR324*, and *Marinisomatota* are the predominant NO reducers by relative abundance. For the 71 N_2_O reducers, 50 (70%) carry *nosZ* only (Fig. 5A), and of the *nosZ* multi-gene denitrifiers (21 MAGs), the majority (17 MAGs) carry *nosZ* with *napA* or *narG*. MAGs carrying *nosZ* account for up to 2% of the community while in metagenomes *nosZ* reaches about 15% of the community. *Marinisomatota* comprise a large fraction of N_2_O reducers by relative abundance, with smaller contributions by *SAR324*, *Actinobacteria*, and other phyla (Figs. 5A, 6A and Supplementary Figs. S6A, S7).

While *nirK*-carrying nitrifiers are generally not considered canonical denitrifiers, we include *nirK* MAGs within the denitrifying community as they can reduce NO_2_^–^ and potentially contribute to loss of fixed nitrogen. NO_2_^–^ reducers carrying *nirK* account for 56 MAGs and up to 6% of the population by relative abundance at the top of the ODZ and in the oxycline, but drops to 2% within the deeper ODZ (Fig. 6A and Supplementary Figs. S6A, S7). This pattern is also reflected within SCG-normalized gene counts in the metagenome assemblies, in which *nirK* reaches up to 40% of the SCG count at 100 m but about 10% below 100 m (Fig. S4A). The most abundant *nirK* containing organisms within our MAGs belong to *Nitrospinota, Thermoplasmatota*, and *Thermoproteota*, while *nirS* nitrite reducers are largely *Proteobacteria* and *Planctomycetota* (Fig. 6A and Supplementary Figs. S6A, S7). The relative abundances of *nirS* MAGs reach only 0.5% in the ETSP and ETNP (Fig. 6A and Supplementary Fig. S6A), but almost 1% in the Arabian Sea (Fig. S7). We find only 9 *nirS* single-step denitrifiers (35%), compared to 34 (61%) *nirK* (Fig. 5A). Out of 26 total *nirS* MAGs, 11 also carry *napA*, 3 carry *narG*, and 10 carry *nor*. However, with the exception of *nirS* denitrifiers, few overlaps exist between denitrifiers carrying genes for NO_2_^–^ reduction, NO reduction, and N_2_O reduction, with most denitrifiers specializing in only one of these steps (Fig. 5 and Supplementary Fig. S10). While MAG incompleteness may contribute to these numbers, we find a similar pattern of single-step denitrifiers when restricting our analysis to MAGs over 90% complete (Fig. S5).

Out of 383 denitrifying MAGs, we obtained only a single complete denitrifier belonging to *Alphaproteobacteria* carrying *napA, nirS*, a noncanonical *nor*, and *nosZ*. This MAG was annotated as genus GCA-2731375 by GTDB, with NCBI taxonomy matching *Rhodospirillaceae*. While this MAG was recovered from the 2018 ETNP ODZ core, similar MAGs within 99% similarity were recovered from 2016 ETNP oxycline and ODZ metagenomes. Similar MAGs from oxycline samples contained only *nor* and no other denitrification genes, but 3 additional ODZ core MAGs assigned to the same genus harbored nearly complete denitrification pathways.

### Other nitrogen cycling phyla, relative abundances, and distributions

MAGs encoding *nxrA*, the nitrite oxidoreductase gene for oxidizing NO_2_^–^ to NO_3_^–^, accounted for 111 MAGs, 55 of which carried only *nxrA* (Fig. S5B). 17 MAGs harbored *nrfA*, and 6 MAGs from the Arabian Sea had co-occurring *nxrA* and *nrfA*. Other nitrogen cycling genes examined, *nifH*, *hzo*, and *amoA*, did not tend to co-occur with denitrification genes in MAGs (Fig. 3 and Supplementary Fig. S5B), although 2 *Candidatus* Scalindua MAGs carrying *hzo* also carried *nirS*, in line with previous reports of anammox *nirS* [3]. These *Ca*. Scalindua MAGs lack a *nor* gene, but two *Ca*. Scalindua MAGs have the *nod* gene for NO dismutase. Relative abundances reveal that *nxr* MAGs comprise up to 8% of the ODZ community, *nrfA*-carrying MAGs up to 6%, and *nifH* and *amoA* MAGs up to around 3% each (Fig. 6B and Supplementary Figs. S6B, S7). In the ETSP and ETNP, *hzo* MAGs comprise up to 4 and 8% of the ODZ community (Fig. 6B and Supplementary Fig. S6B), but only about 3% in the Arabian Sea (Fig. S7). The single-copy-gene-normalized metagenome data largely supports these trends, although a higher percentage of *nxrA* genes (up to 40%) compared to *hzo* genes (up to 15%) were found within metagenomes (Fig. S4B). While surface metagenomes show a near-zero count of denitrification and most other nitrogen-cycling genes, *nifH* counts peak at the surface and quickly drop to near zero below 100 m depth. As few of our MAGs were assembled from or mapped to surface metagenomes (Fig. S3), our MAG-based estimates of *nifH* underestimate the number and abundance of nitrogen fixers.

The majority of the *nxrA* community in the ETNP and ETSP are *Planctomycetota*, with smaller fractions contributed by *Nitrospinota, Marinisomatota*, and *Chloroflexota*. 16 Marinisomatota MAGs carried *nxrA*, with 4 MAGs carrying both *nxrA* and *narG*. The *nrfA* community is almost entirely dominated by *Marinisomatota* by relative abundance. Similarly, *amoA* is dominated by *Thermoproteota*, ammonia-oxidizing archaea (AOA). Nitrogen fixers carrying *nifH* in the upper depths of the ODZ and the oxycline are primarily Cyanobacteria, while deeper depths are dominated by heterotrophic *Proteobacteria* and *Actinobacteria*. As expected, all *hzo* genes in our dataset belong to *Planctomycetota* assigned to *Ca*. Scalindua, the dominant ODZ anammox organism, which despite numbering only 5 MAGs in this set comprises up to 4%, 9%, and approximately 3% of relative abundance in the ETSP, ETNP, and Arabian Sea ODZ communities, respectively (Fig. 6B and Supplementary Figs. S6B, S7).

Overall relative abundances of nitrogen cycling genes, calculated using both SCG-normalized metagenome hits and MAG relative abundance mapping, follow similar patterns. The potential for NO_3_^–^ reduction to NO_2_^–^ dominates across almost all ODZ depths (Fig. 5B and Supplementary Figs. S4, S10). The next largest contribution is potential *nor*-mediated NO reduction to N_2_O. However, *nirK*-catalyzed NO_2_^–^ reduction potential peaks between 50–100 m in the oxycline and upper ODZ, but forms a much smaller proportion below 100 m (Figs. S4A, S10). While contributions of *amoA* and *nifH* to the ODZ community are smaller, the genes and organisms are not absent (Fig. 6B and Supplementary Figs. S6B, S7). MAGs harboring the *nrfA* gene are consistently present across ODZ depths. The potential for NO_2_^–^ oxidation to NO_3_^–^ mediated by *nxr* appears substantially in both metagenomes and MAGs, and is widespread across MAGs.

### Chemotaxis and motility genes in denitrifying MAGs

**Fig. 7:**
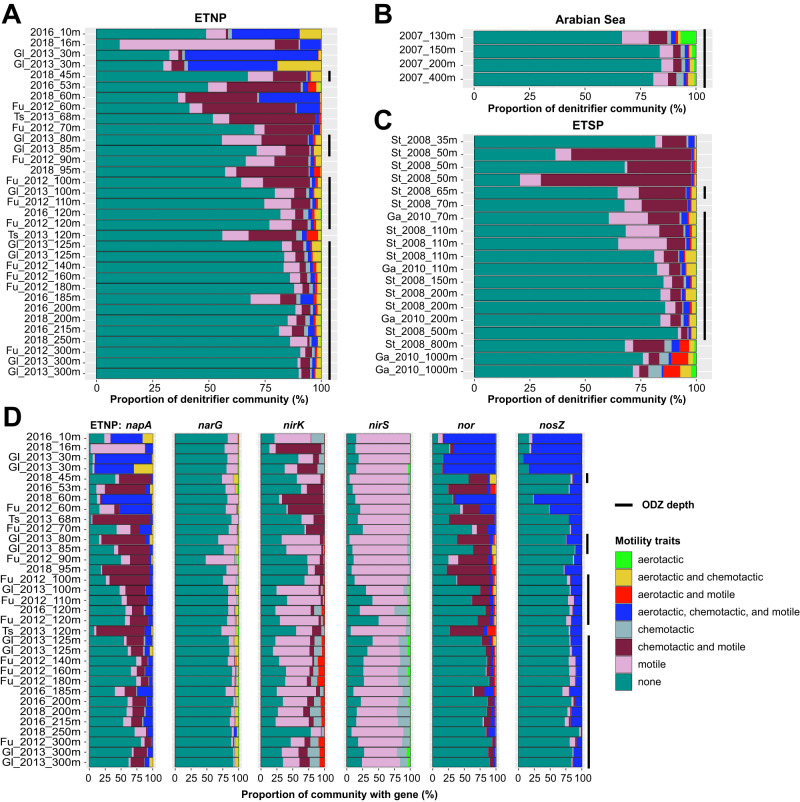
Motility and chemotaxis characteristics of denitrifier MAGs. Relative abundances of denitrifier MAGs across all metagenomes in the (**A**) ETSP, (**B**) Arabian Sea, and (**C**) ETNP ODZs, color-coded by presence of motility, chemotaxis, and aerotaxis genes. Relative abundances of non-denitrifying MAGs showing the same traits can be found in Supplementary Fig. S9A. **D** Relative abundance mapping of all MAGs carrying each denitrification gene across all ETNP metagenomes. MAGs are included if the denitrification gene in question is present, regardless of the presence or absence of other genes. MAGs are colored by the presence of motility, chemotaxis, and aerotaxis genes. Black bars to the right of each graph indicate ODZ depths (O_2_ < 3 μM). Mapping results for ETSP metagenomes can be found in Supplementary Fig. S9.

Searching for motility and chemotaxis-related genes within MAGs reveals a higher proportion of these traits within denitrifiers compared to non-denitrifiers (Fig. 7A and Supplementary Fig. S9A). Within 383 denitrifying MAGs, 108 (28%) possessed chemotaxis, motility, or aerotaxis capability, compared to 19% of non-denitrifiers. Comparing denitrifiers to non-denitrifiers, 20% vs. 12% were motile, 14% vs. 5% chemotactic, and 8% vs. 2% aerotactic. MAGs with both chemotaxis and motility genes were particularly prevalent in the denitrifying community, comprising 20–50% in the oxycline and upper ODZ (Fig. 7A), while at the same depths they are less than 5% of the non-denitrifying community (Fig. S9A). Less than 1% of non-denitrifiers have both aerotaxis and chemotaxis or aerotaxis and motility, but 5–20% of denitrifiers have these traits at ODZ depths, with an even higher percentage in the oxycline. However, motility without chemotaxis or aerotaxis appears widespread among non-denitrifiers (Fig. S9A).

Motile and chemotactic MAGs are in highest abundance within the *napA* community, making up the majority at oxycline and upper ODZ depths (Fig. 7B and Supplementary Fig. S9B). However, they make up very little (<1%) of the *narG* community, which is primarily dominated by non-motile organisms. Motile or chemotactic MAGs also comprise a sizeable fraction of *nirK, nirS, nor*, and *nosZ* communities, with *nirK* and *nirS* dominated by motile but not chemotactic or aerotactic MAGs, *nor* by motile MAGs with chemotaxis and/or aerotaxis, and *nosZ* by MAGs with aerotaxis, chemotaxis, and motility. With the exception of *nxrA* and *nod*, communities with the other nitrogen cycling genes are primarily non-motile, non-chemotactic, and non-aerotactic (Fig. S9C, D).

## Discussion

### Unique ODZ microbial communities reflected by the largest MAG collection

Sequencing efforts devoted to open ocean oxygen-deficient zones have revealed much about the microbial community therein, including marked shits in community structure from oxic surface waters to the anoxic core [31, 51], diverse, atypical denitrification genes [9, 15], distinct particle-associated and planktonic assemblages [9, 31, 32], and a large number of uncultured organisms with surprising metabolic capabilities [38, 41, 85, 86]. ODZs, due to their distinctive chemical profiles compared to the oxygenated ocean, harbor unique, largely uncultivated microbial communities [87, 88]. Our 962 MAGs present the largest collection of genomes from global, open-ocean ODZs to date. The low mapping rate of our ODZ MAGs to surface metagenomes indicate a resident microbial community within the ODZ distinct from that of the surface ocean (Fig. S3). This is consistent with previous studies using ODZ metagenomes [9, 31, 41, 51] as well as 16 S amplicon sequencing [31].

The increase in relative abundance of anaerobic nitrogen metabolism genes, such as those involved in denitrification and DNRA, from the surface to ODZ depths indicates adaptation of the microbial community to low-oxygen conditions. While previous metagenomics efforts have uncovered the identities of specific nitrogen cycling taxa [38, 41–43], our approach takes a broader view of the nitrogen cycling community across multiple ODZs to reveal a stable denitrifying community dominated by several phyla. *Proteobacteria*, one of the most abundant phyla across ODZ depths, include a wide range of *Alphaproteobacteria* and *Gammaproteobacteria* known to participate in heterotrophic denitrification [31]. Another abundant ODZ phylum is the *Marinisomatota*, also known as Marine Group A, *SAR406*, or *Marinimicrobia* [38]. Despite being widespread in anoxic marine environments, little is known about the metabolism and ecophysiology of this uncultivated clade [38]. However, two *Marinisomatota* MAGs from anoxic waters of the Costa Rican Golfo Dulce were reported to contain and actively transcribe *nar* genes for NO_3_^–^ reduction [38], and a separate study of the ETSP found NO_3_^–^ reductases in 6 out of 8 *Marinisomatota* MAGs, with 3 of these MAGs in the top 5 most abundant in the collection [45]. While we find diverse nitrogen cycling capabilities within this phylum, including NO_3_^–^ reduction and NO_2_^–^ oxidation, 44 *Marinisomatota* MAGs carry no queried nitrogen cycling genes. While we allude to phyla carrying nitrogen cycling genes of interest, overall, high inter-clade metabolic variability prevents us from assigning metabolic capabilities to specific taxa.

### Niche differentiation of MAGs carrying single denitrification genes

Despite co-occurrence among the denitrification genes *napA, narG, nirS, nor*, and *nosZ*, these genes are carried by different microorganisms rather than within the genomes of complete denitrifiers. Although we identified only a single complete denitrifier, this does not preclude the existence of complete denitrifiers in this environment since MAGs vary in completeness and may not capture the entire genome, and not all organisms assembled into MAGs. However, the presence of many partial denitrifiers with over 90% completeness (Fig. S4A) and disparity between relative abundances of different denitrification genes in metagenomes (Fig. S4A) point to the rarity of complete denitrifiers in the ODZ water column, although particle associated denitrifying communities may be distinct. Our results corroborate previous reports of partial denitrifier prevalence in agricultural and tundra soils [36, 89] as well as within a small number of previously described ETSP ODZ MAGs [45].

While NO_3_^–^ reducers carrying *narG* and those carrying *napA* co-occur across depths, these genes do not frequently co-occur within genomes and are largely carried by different phyla. A third nitrate utilization gene, the assimilatory nitrate reductase encoded by *nas*, also contributes to NO_3_^–^ and NO_2_^–^ cycling by converting these substrates to ammonium for incorporation into biomass. However, organisms with *nas* alone are generally excluded from canonical nitrogen oxide reducers, and thus we focus on dissimilatory metabolisms. *SAR324*, formerly classified within the *Deltaproteobacteria* but recently reclassified into its own phylum, is the most abundant *napA* NO_3_^–^ reducer, but we found no *narG* genes within *SAR324* MAGs in our collection. *SAR324* is a diverse, uncultivated clade with wide oceanic distributions, particularly in low-oxygen zones [90]. *SAR324* genomes from a hydrothermal plume carried *nosZ* and *nirK* [91], and *SAR324* from coastal and deep sea sediments were found to encode for diverse nitrogen cycling genes including *napA* and *nosZ* [92]. We find *SAR324* to comprise 30% or more of *napA* NO_3_^–^ reducers, *nor* NO reducers, and *nosZ* N_2_O reducers within ODZ depths by relative abundance, totaling 8, 13, and 5 MAGs each, respectively. All our *SAR324* MAGs belong to the genera *Arctic96 AD-7, UBA1014*, or *UBA8110*, with a patchy distribution of nitrogen cycling capabilities throughout clades. No *nap* or *nar* genes were discovered within 8 MAGs in our collection assigned to the *SAR11* family *Pelagibacteraceae*, despite previous reports of *SAR11* NO_3_^–^ reducers [43]. However, high genomic diversity and frequent recombination in *SAR11* populations poses challenges for generating high-quality *SAR11* MAGs [93], and only 3 of our *SAR11* MAGs are >70% completion. Our MAG-based analysis likely underrepresents ODZ *SAR11* abundance and diversity, and this may contribute to the higher relative abundance of *nap/nar* in metagenomes compared to the relative abundance of NO_3_^–^ reducer MAGs.

Although the enzymes encoded by *napA* and *narG* perform the same reduction process, the *napA* periplasmic nitrate reductase is not thought to provide energy for cells via a proton motive force, whereas the *narG* membrane-bound nitrate reductase does [94]. NapA has been implicated in balancing the intracellular redox state under oxygen limitation [95], and has been reported to reduce NO_3_^–^ under aerobic conditions and hold a selective advantage over NarG under limiting NO_3_^–^ conditions [94, 96]. As NapA does produce NO_2_^–^ which can be used by Nir, there is observable overlap between *napA* and *nirS*. Our results imply niche differentiation between these two types of NO_3_^–^ reducers. High NO_3_^–^ concentrations in ODZs may enable co-existence of these NO_3_^–^ reducer ecotypes, particularly since NO_3_^–^ is never depleted in the bulk ocean. Furthermore, abundant *napA* NO_3_^–^ reducers frequently carry motility and chemotaxis genes, which may facilitate particle colonization [34, 35, 97] and suggests a particle-associated lifestyle, whereas dominant *narG* NO_3_^–^ reducers are primarily non-motile (Fig. 7B and Supplementary Fig. S9B). The lack of motility among *narG*-containing organisms suggests a more planktonic lifestyle whereby these organisms are sustained by dissolved organic and inorganic nutrients. Moreover, previous studies [9, 31] report that *narG* is more frequently contained among planktonic cells rather than particle-associated size classes.

Niche differentiation appears between *nirS* and *nirK* nitrite reducers (Fig. 6A and Supplementary Fig. S6A). MAGs carrying *nirK* genes belong largely to ammonia oxidizing archaea (AOA) or nitrite oxidizing bacteria (NOB) involved in the nitrification pathway, including *Nitrospinota*, the dominant marine NO_2_^–^ oxidizer [98]. We find that *nirK* may predominate within nitrifiers. In line with previous reports [99], *nirK* does not co-occur with *nor* in ODZ AOA (Fig. 5A), and the role of *nirK* in these organisms remains debated [99, 100]. As NO is an essential intermediate for AOA ammonia oxidation [101], NO release by NOB has been hypothesized to promote mutualistic interactions between NOB and AOA [85]. In contrast, *nirS* may predominate within heterotrophs that are more commonly multi-step denitrifiers. However, *nirS* MAGs are less abundant overall and *nirS* is also found within anammox bacteria belonging to *Ca*. Scalindua. This is in line with a previous amplicon-based survey from similar sampling sites in the ETNP and Arabian Sea, indicating low *nirS* abundance and diversity and high similarity between the most abundant *nirS* OTU and *Ca*. Scalindua *nirS* [102]. Although *nirS* and *nirK* have been used as functional markers for denitrification [103, 104], these genes cannot be viewed interchangeably and may not reflect the activity of upstream or downstream denitrification steps.

The majority of *nir* MAGs lack *nor* (Fig. 5), despite the toxicity of the resultant NO. Yet nearly half of all *nirS* MAGs contain *nor* or *nod*, in contrast to *nirK* MAGs. Diverse, non-canonical *nor* genes have recently been discovered [7, 8, 66] and comprise the majority of *nor* genes we identified. Few *nir*-possessing MAGs also carried the NO dismutase gene *nod*, indicating novel NO detoxification methods likely exist within environmental microorganisms beyond canonical *nor* and *nod*. The *nirK*-encoded nitrite reductase in nitrifiers has been suggested to catalyze the oxidation of NO to NO_2_^–^ [105]. Alternatively, NO may act as an intercellular signaling molecule to modulate the behavior of interaction partners [106, 107]. The prevalence of *nor* genes, particularly single-step *nor* MAGs, may indicate a widespread need to detoxify NO despite the low concentrations of NO in bulk seawater [108], potentially as a result of NO secretion from spatially proximate neighbors such as within a particle. The diversity and function of non-canonical *nor* genes within ODZs has not previously been reported and warrants further exploration.

The prevalence of partial denitrifiers carrying only *nosZ* indicates potential for ODZ microorganisms to act as a sink for N_2_O, and supports previous work showing substantial consumption of N_2_O in oxyclines and ODZs [109] and rapid turnover of N_2_O [21]. However, the nanomolar concentrations of N_2_O, its rapid diffusion, and the low oxygen tolerance of N_2_O reductase poses challenges for denitrifiers relying solely upon *nosZ*. We find the motile fraction of *nosZ* MAGs to be dominated by MAGs with genes for aerotaxis, chemotaxis, and motility, comprising up to 30% of the *nosZ* community at ODZ depths (Fig. 7 and Supplementary Fig. S9). The *aer*-encoded aerotaxis receptor senses oxygen gradients, and has been reported to facilitate the movement of organisms both towards and away from oxygen [83, 110]. Along with chemotaxis, this may enable organisms to seek out localized regions of high carbon and N_2_O and navigate oxygen gradients, such as those found within particles or at the oxycline/ODZ interface. Previous metagenomic analyses of size-fractionated ETNP communities [9] found higher abundances of genes for the last two steps of denitrification on particles compared to free-living communities. Our dataset, while not specifically targeting particles, does not exclude particles from sampling, as we find a number of motility and chemotaxis genes associated with particle colonization while planktonic communities are predominantly nonmotile [35]. Denitrification in particles is an active area of research, and has been posited to expand the niche of anaerobic metabolisms [33]. The higher abundance of motile and chemotactic denitrifiers, particularly ones carrying *nir, nor*, and *nosZ*, compared to non-denitrifiers (Fig. 7 and Supplementary Fig. S9), supports the importance of particle-based denitrification and opens up fruitful avenues for further research into particle colonization and metabolisms.

The presence of 29 bacterial MAGs with aerotaxis or chemotaxis but not flagellar motility may result from MAG incompleteness, but the average completeness of 83% for these MAGs suggests the possibility of alternative motility mechanisms or functions of the chemosensory system. Potential interplay of the chemotaxis machinery has been described with pili-mediated surface motility, cell aggregation, virulence, and biofilm formation [111]. Further characterization of chemotaxis, aerotaxis, and motility in marine microorganisms presents an exciting route for future work.

### Other nitrogen cycling genes and the NO_3_^–^ ⇆ NO_2_^–^ loop

We find NO_2_^–^ oxidation potential in a wide diversity of phyla, yet only MAGs belonging to phyla *Marinisomatota, Nitrospinota*, and *Planctomycetota* reach relative abundances over 1% of the community (Fig. 6B and Supplementary Figs. S6B, S7). NO_2_^–^ oxidation has been described as an aerobic process, but requires O_2_ only as a terminal electron acceptor [112]. *Nitrospinota* have been discovered in ODZ waters and postulated to evolve from microaerobic or anaerobic ancestors [41, 98]. The prevalence of NO_2_^–^ oxidation and discovery of *Nitrospinota* in ODZs has led to hypotheses of fully anaerobic *Nitrospinota* that use terminal electron acceptors other than oxygen for NO_2_^–^ oxidation. However, consistent with previous studies [113, 114], we find *Nitrospinota* dominant within *nxr*-carrying MAGs only at oxycline and upper ODZ depths (Fig. 6B and Supplementary Fig. S6B), where they may be sustained by cryptic oxygen cycling in the secondary chlorophyll maximum [114] or ephemeral intrusions of oxygenated water [49, 115]. Within permanently anoxic ODZ depths, *nxr*-carrying *Planctomycetota* comprise the most abundant NO_2_^–^ oxidizer, including MAGs belonging to the anammox bacterium *Ca*. Scalindua. The occurrence of *nxr* in anammox bacteria is well established [116, 117], and thought to harvest electrons for the reduction of NO_2_^–^ to NO, an important intermediate in the anammox pathway. As high rates of NO_2_^–^ oxidation have been found in both oxycline and anoxic ODZ waters [118], microaerobic NO_2_^–^ oxidation by *Nitrospinota* and anaerobic NO_2_^–^ oxidation by anammox bacteria may both contribute to the NO_3_^–^ ⇆ NO_2_^–^ loop within ODZs.

The *nxr* gene co-occurs within MAGs along with various denitrification genes and the DNRA gene *nrfA* (Fig. S5B). The heterogeneous availability of organic carbon may confer a benefit to a mixotrophic lifestyle wherein microorganisms partition NO_2_^–^ to different metabolisms depending on the environmental situation. Previous work in the ETSP and Arabian Sea has uncovered DNRA [3, 119], although a link between *nrfA* marker genes and taxonomic identity was not possible. We find *nrfA* distributed across bacterial phyla, but only *Marinisomatota* as an abundant DNRA organism in all ODZs and depths in which *nrfA* was present (Fig. 6B and Supplementary Figs. S6B, S7).

Previous studies have discussed the fate of NO_2_^–^ and whether it is primarily oxidized back to NO_3_^–^ or further reduced via downstream denitrification, anammox, or DNRA. Recent work using isotope measurements [17, 120] and proteomics [121] indicate a large contribution of NO_2_^–^ oxidation within anoxic waters. Our results are in line with previous findings, but also reveal a large diversity in the NO_2_^–^ utilization metabolisms in the ODZ and the taxa potentially performing them. Based upon MAG relative abundance and normalized marker gene abundance within metagenomes, we present a picture of nitrogen cycling potential across the ODZ (Fig. 1). We find a diversity of nitrogen metabolisms with the key intermediate NO_2_^–^ partitioned amongst anammox, DNRA, and denitrifying microorganisms in the ODZ, along with a large nitrifier contribution in the oxycline. The relative dominance of these metabolisms may be driven by competition for carbon along with bioavailable nitrogen, as well as enzyme tolerances for oxygen and interspecies interactions. The modularity of denitrification genes, and possibly other nitrogen cycling genes, may enable organisms to acquire these metabolisms as needed via mechanisms such as horizontal gene transfer. Metagenomics may not reliably reflect the activity of a pathway, as the possession of a gene does not necessitate its active transcription and function, and the regulation of denitrification genes in partial denitrifiers remains to be fully comprehended. However, we find significant correlations between the rates of nitrogen transformations and their encoding genes. The broad patterns we find in gene content represent a metabolic potential present within microorganisms across ODZs and depths and reflect the adaptive processes shaping these communities.

Much remains to be discovered about nitrogen cycling within the ODZ and the communities performing these metabolisms. Importantly, integrating genomic information from MAGs with biogeochemical tracers and reaction rates for nitrogen cycling processes is a necessary step to identify the major microbial players in this system, resolve their activities in the water column, and predict how these communities will respond to and shape the global nitrogen cycle under changing climate conditions. Further studies on the full range of sequence space for given nitrogen cycle proteins require a closer look into microbial physiology and metabolism in environmental microorganisms, possibly through culture efforts targeting understudied ODZ taxa.

## Supplementary Information

Supplementary Information

Supplementary Dataset 1

Supplementary Dataset 2

Supplementary Dataset 3

## Data Availability

MAGs and new metagenome assemblies from this study are available from NCBI under BioProject ID PRJNA955304. A full list of MAGs and accession numbers can be found in Supplementary Dataset 1.

## Code availability

Code for metagenome assemblies, MAG generation, and bioinformatics analyses are available on GitHub at https://github.com/izhang73/ODZ-MAGs. Custom Hidden Markov Models and seed sequences used to generate HMMs are available on Github.
