# Supplementary material for: Partitioning of the denitrification pathway and other nitrite metabolisms within global oxygen deficient zones
Source: ISME Commun. 2023 Jul 20;3:76. doi: 10.1038/s43705-023-00284-y (PMC10359470; doi:10.1038/s43705-023-00284-y)
Supplement: Supplementary file 1 — Supplementary Information [file 43705_2023_284_MOESM1_ESM.pdf]

Supplementary Information for

Partitioning of the denitrification pathway and other nitrite metabolisms within global oxygen deficient zones

Authors: Irene H. Zhang[1,2*], Xin Sun[3,4], Amal Jayakumar[4], Samantha G. Fortin[4], Bess B. Ward[4], and Andrew R. Babbin[1*]

[1]Department of Earth, Atmospheric and Planetary Sciences, Massachusetts Institute of Technology, Cambridge, MA

[2]Program in Microbiology, Massachusetts Institute of Technology, Cambridge, MA

[3]Department of Global Ecology, Carnegie Institution for Science, Stanford, CA

[4]Department of Geosciences, Princeton University, Princeton, NJ

*Corresponding authors: izhang@mit.edu and babbin@mit.edu

Contents

Supplementary Table S1

Supplementary Figures S1–S10

Supplementary Datasets S1–S3

Reference	NCBI ID	Latitude (°N)	Longitude (°E)	Year	Ocean Basin	Cruise & Station Number (if known)
This study	PRJNA955304	18.7	-104.4	2016	ETNP	RB-16-03<br>PS-S6
This study	PRJNA955304	17.2	-110.7	2016	ETNP	RB-16-03<br>14
This study	PRJNA955304	15.2	64	2007	Arabian Sea	KNOX009<br>2N
This study	PRJNA955304	16	-105	2018	ETNP	SR1805<br>PS2
This study	PRJNA955304	18	-102	2018	ETNP	SR1805<br>PS3
Fuchsman et al. (2017)	PRJNA350692	17	-106.5	2012	ETNP	TN278<br>136
Fuchsman et al. (2017)	PRJNA350692	16.5	-107.1	2012	ETNP	TN278<br>BB2
Glass et al. (2015)	PRJNA254808	18.9	-108.8	2013	ETNP	NH-1315<br>2
Glass et al. (2015)	PRJNA254808	18.9	-106.3	2013	ETNP	NH-1315<br>4
Glass et al. (2015)	PRJNA254808	18.9	-104.5	2013	ETNP	NH-1315<br>6
Glass et al. (2015)	PRJNA254808	18.8	-104.7	2013	ETNP	NH-1315<br>10
Tsementzi et al. (2016)	PRJNA323946	18.5	-104.5	2013	ETNP	OMZoMBiE<br>6
Stewart et al. (2012)	PRJNA68419	-20.1	-70.4	2008	ETSP	MOOMZ
Ganesh et al. (2014)	PRJNA217777	-20	-70.8	2010	ETSP	BiG RAPA

Supplementary Table S1. References, NCBI BioProject IDs, sampling latitude and longitude, sampling year, sampling ocean location, cruise name, and sampling station ID for each set of metagenomes included in this study. A full list of depths and associated oxygen and nutrient profiles can be found in Supplementary Dataset S2.

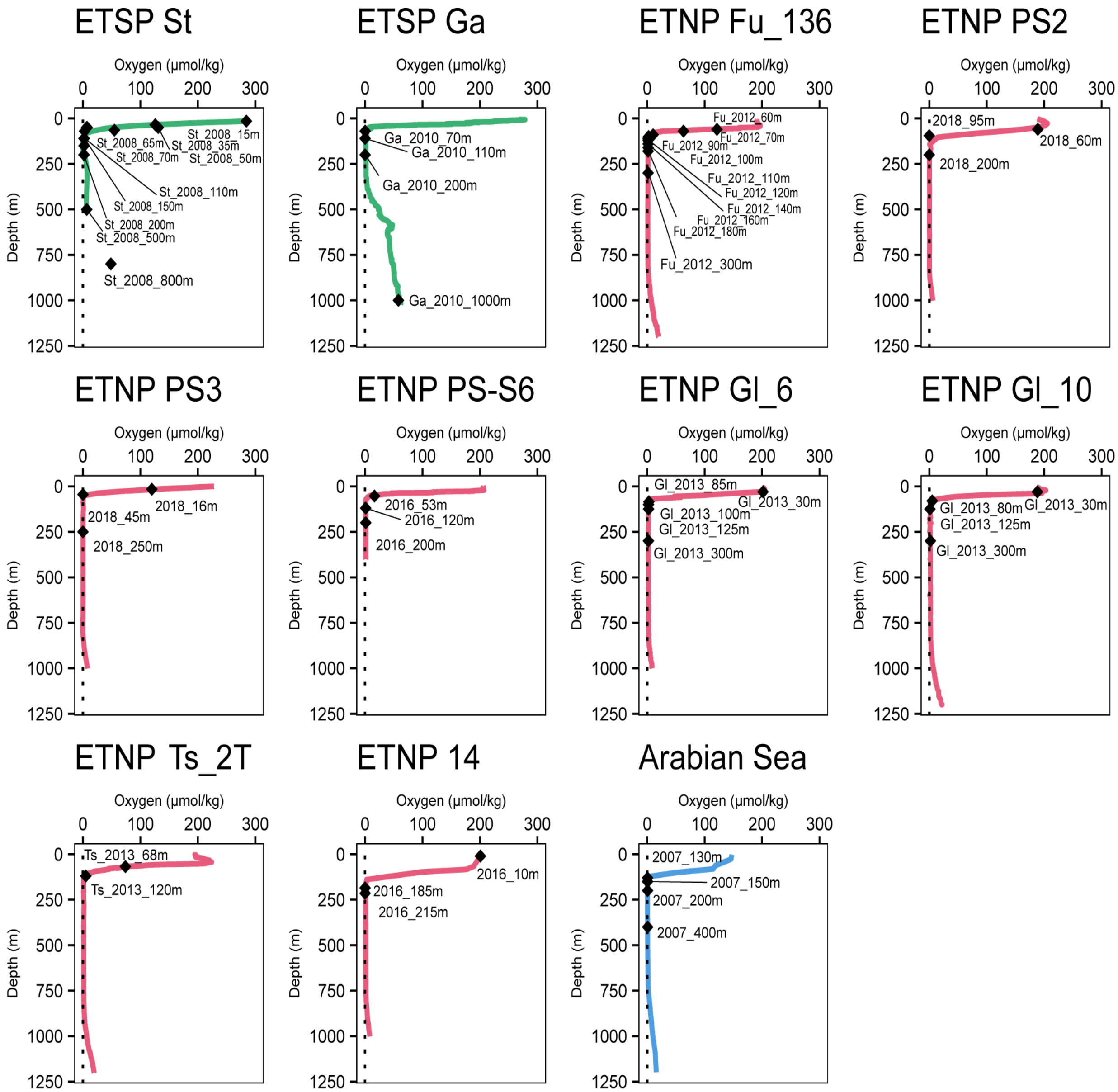

Supplementary Figure S1. Oxygen profiles from CTD casts for relevant sampling stations from which metagenomes were assembled. The naming scheme is as follows: St for Stewart profiles, Ga for Ganesh metagenomes, Fu for Fuchsman profiles, Gl for Glass profiles and Ts for Tsementzi profiles, while letters and numbers following the underscore correspond to sampling sites following the original cruise naming scheme. Only 1 sampling site and profile corresponds to Stewart and Ganesh metagenomes respectively and to Arabian Sea metagenomes. ETNP PS2, PS3, PS-S6, 14, and Arabian Sea are profiles for metagenomes from this study. Black diamonds correspond to metagenome sampling depths, and text labels match the metagenome naming scheme used throughout the paper.

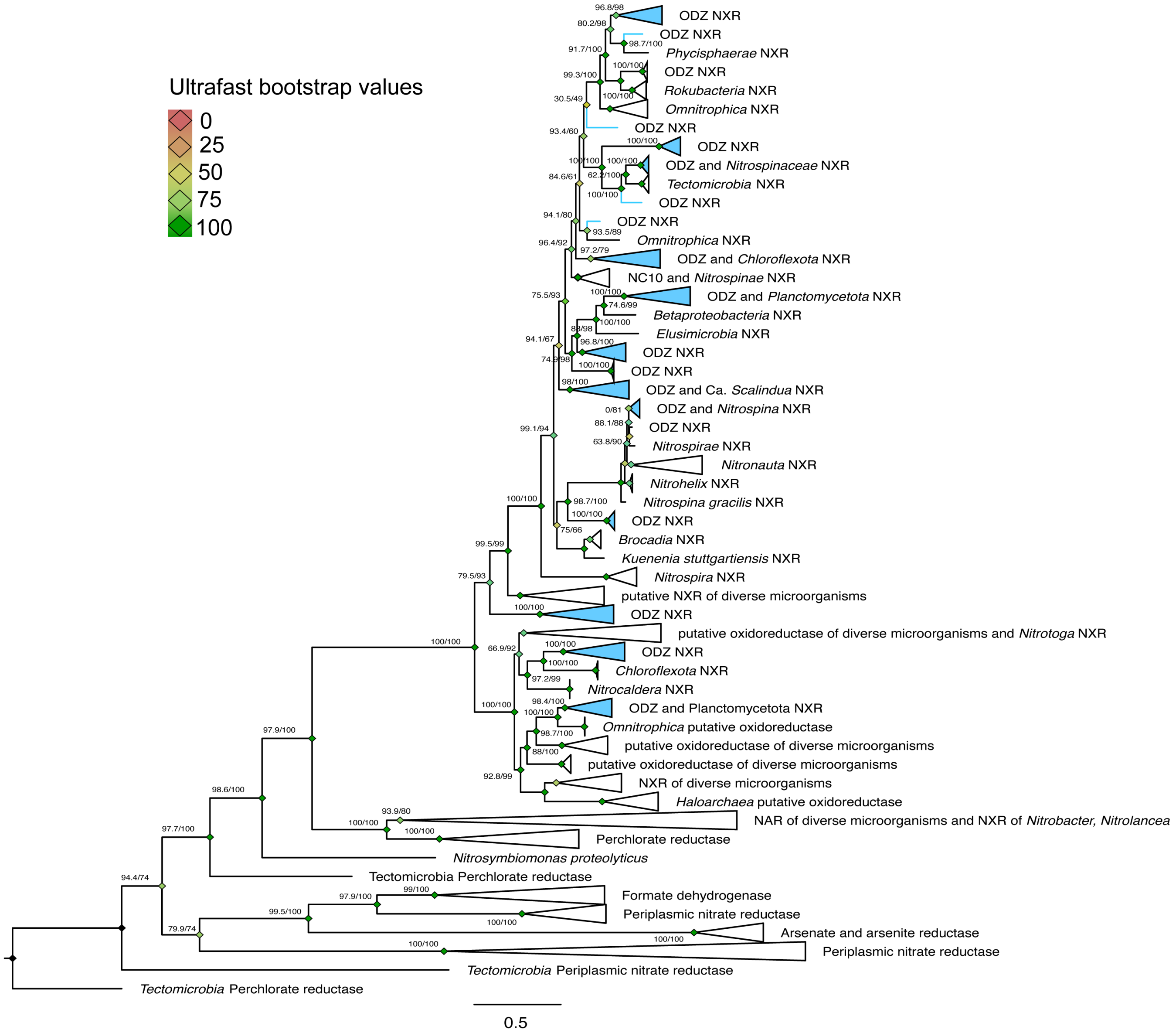

Supplementary Figure S2. Phylogenetic protein tree of nitrite oxidoreductase (NXR) proteins and the related proteins respiratory nitrate reductase (NAR), periplasmic nitrate reductase, formate dehydrogenase, perchlorate reductase, arsenate reductase, and arsenite reductase. NxrA sequences from ODZ MAGs are colored in blue and annotated as ODZ NXR. Node colors correspond to ultrafast bootstrap values, and node labels are the values of the SH-like approximate likelihood ratio test and ultrafast bootstrap values in the format SH-aLRT/UFboot.

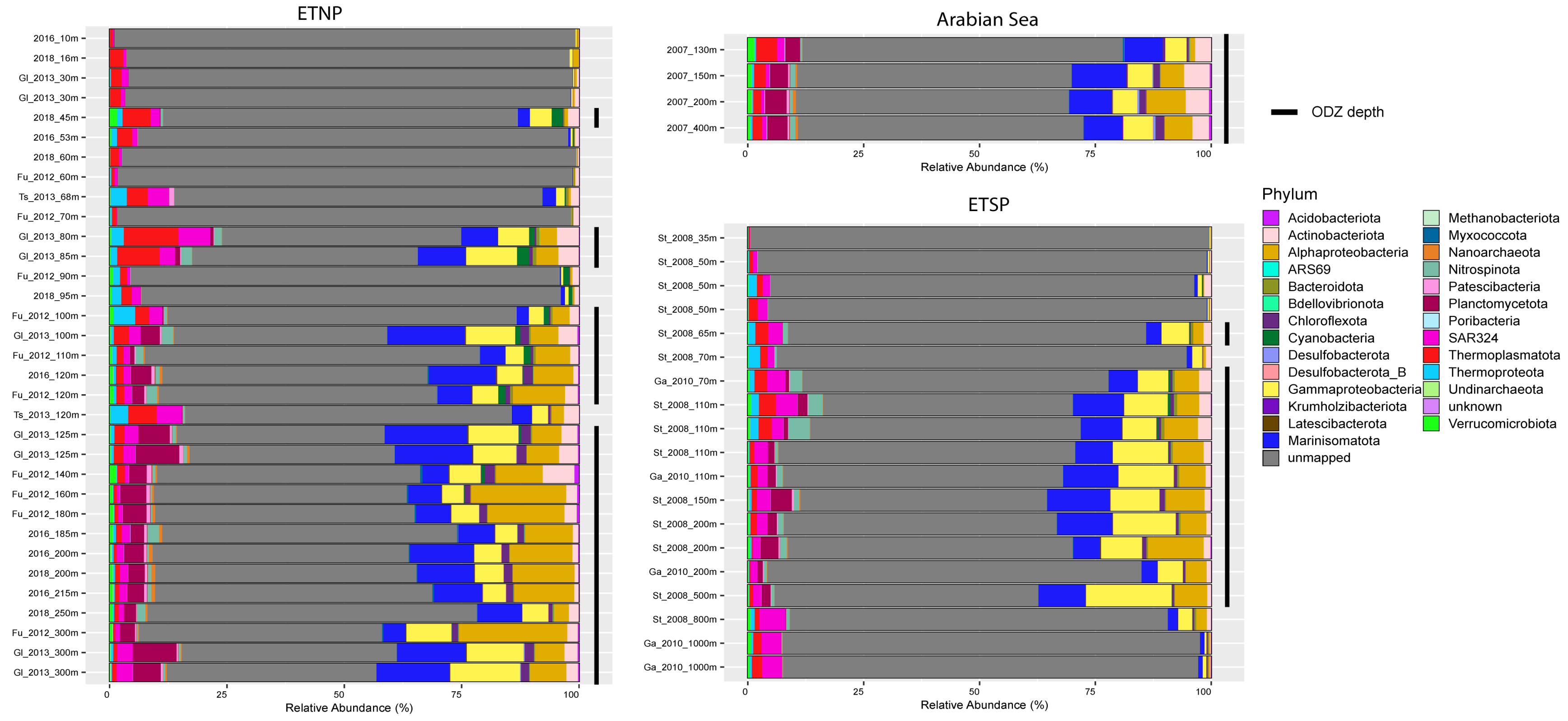

Supplementary Figure S3. Relative abundances of all dereplicated MAGs against all short reads from each metagenome, colored by phylum-level taxonomy, except for *Proteobacteria* which is colored by class. Grey bars indicate reads that were not mapped to any MAG in the collection. Metagenomes are arranged in the same order as in Figure 4A. Black bars above the graph indicate ODZ depths ($O_2 < 3$ μM).

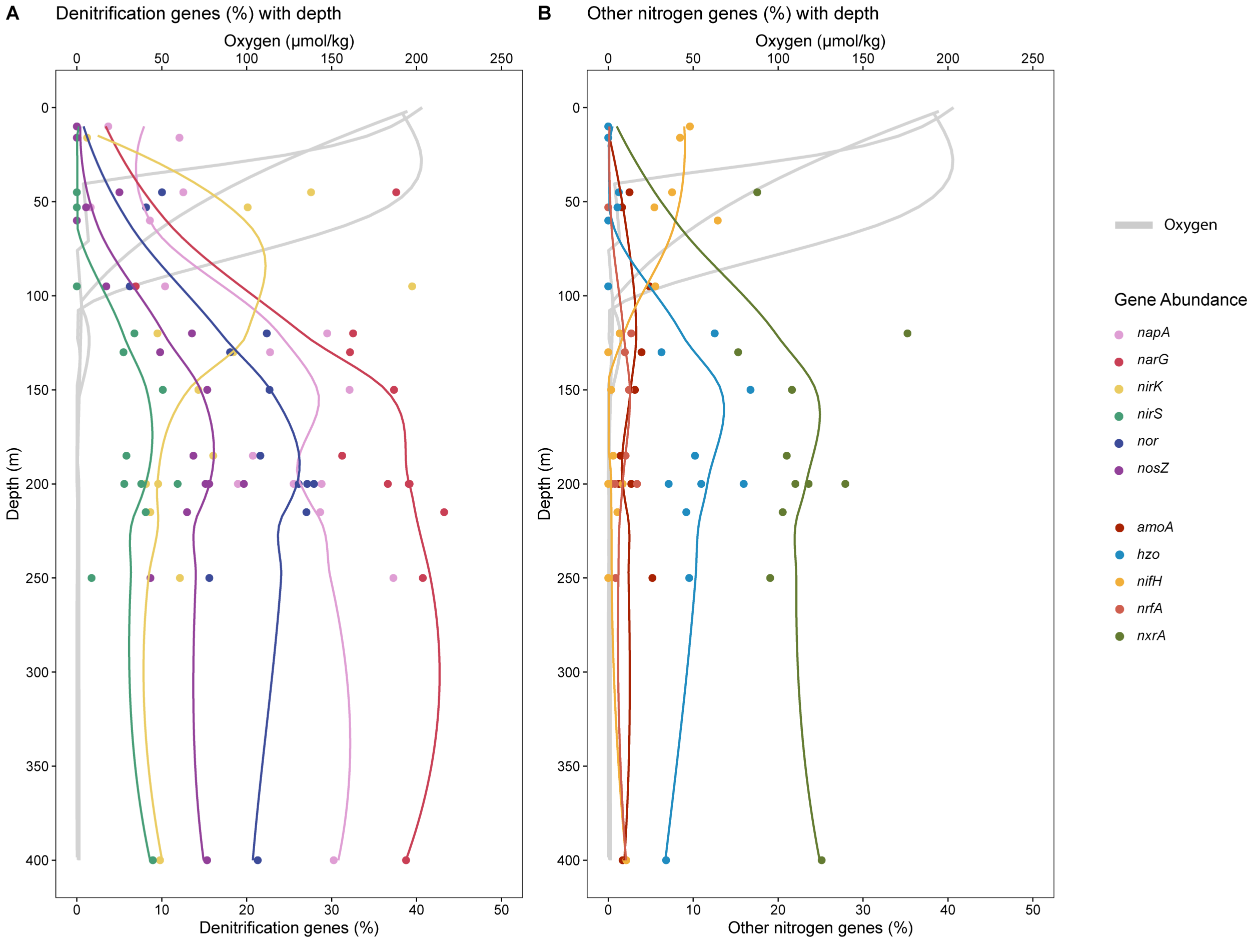

Supplementary Figure S4. A, Relative abundance of hits for each denitrification gene within individually assembled ETNP metagenomes from this study. Relative abundances are normalized against the average number of hits for single-copy genes *rpoB, rplB, gyrB, recA*, and *rpS3*. Smoothed oxygen profiles for the same sampling sites are depicted in grey. **B**, Relative abundance of hits for each non-denitrification nitrogen cycling gene within individually assembled ETNP metagenomes from this study, calculated as in **A**.

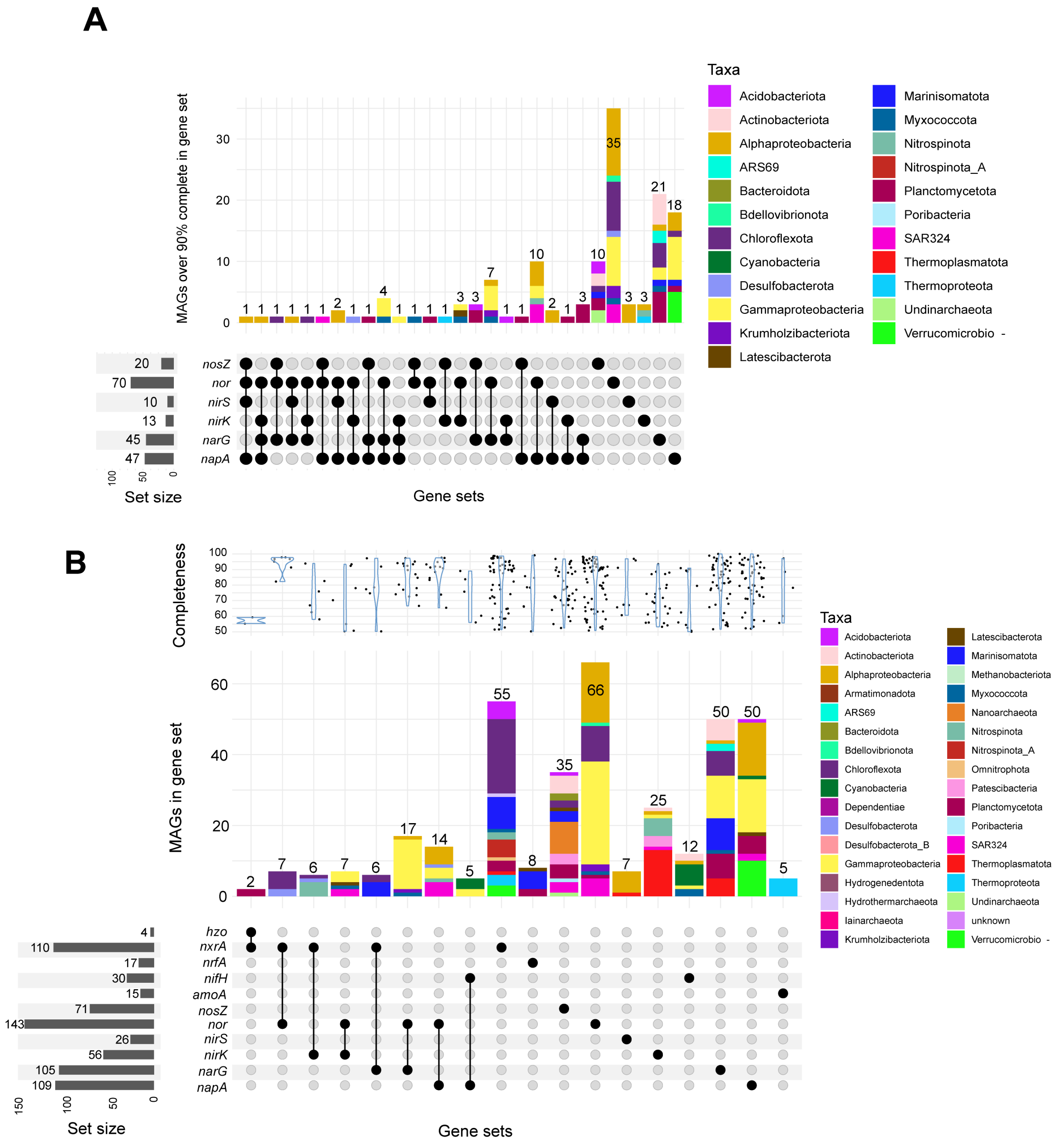

Supplementary Figure S5. A, The number of MAGs carrying each denitrification gene set within the ODZ MAG collection, showing only MAGs over 90% complete. Top panel shows the number of MAGs colored by phylum-level taxonomy, except for *Proteobacteria* which is colored by class. Bottom panel shows the genes within each gene set. Left bottom panel shows the number of MAGs carrying each specific denitrification gene. **B**, The number of MAGs carrying each nitrogen cycling gene set, including denitrification genes, within the ODZ MAG collection. Sets falling below 5 MAGs, with the exception of *hzo*, are omitted for brevity. Top panel shows the completeness (%) and the completeness distribution of the MAGs in that gene set, middle panel shows the number of MAGs colored by phylum or class-level taxonomy, and bottom panel shows the genes within each gene set. Left bottom panel shows the number of MAGs carrying each specific nitrogen cycling gene.

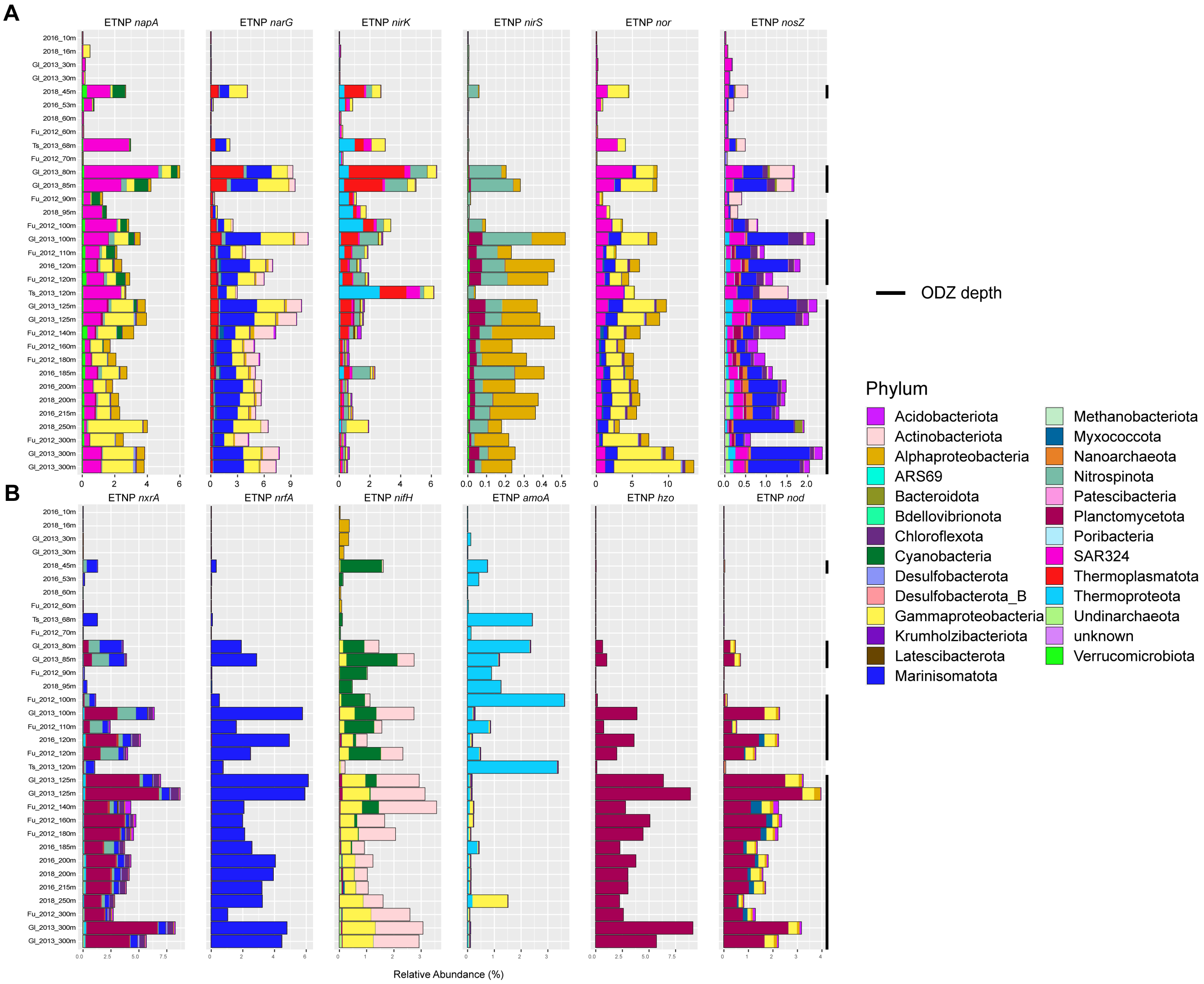

Supplementary Figure S6. Relative abundances of MAGs in the ETNP across all metagenomes carrying each (**A**) denitrification gene or (**B**) other nitrogen-cycling genes. MAGs are colored by phylum-level taxonomy, except for *Proteobacteria* which is colored by class. Black bars above the graph indicate ODZ depths (O_2 < 3 μM).

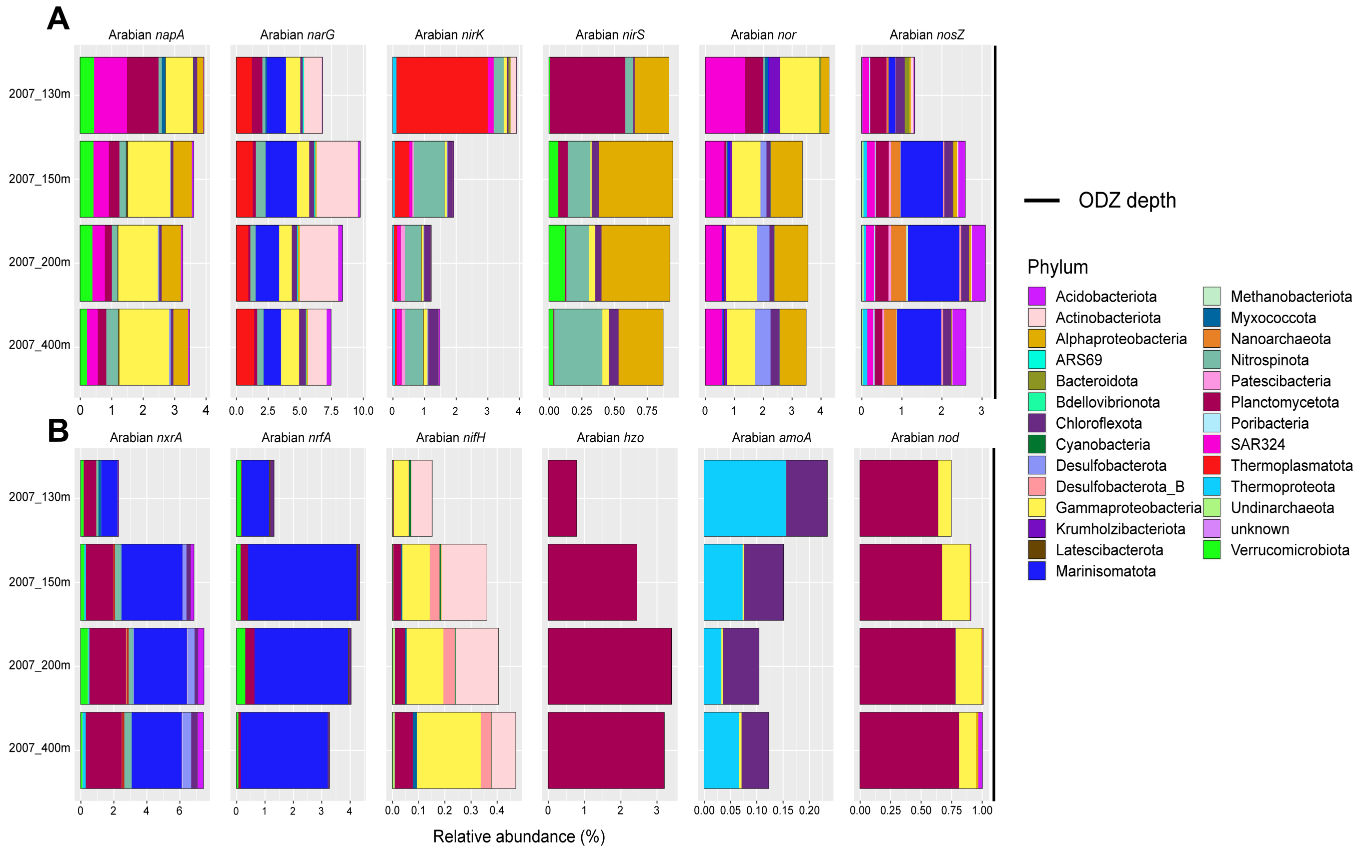

Supplementary Figure S7. Relative abundances of MAGs in the Arabian Sea across all metagenomes carrying each (**A**) denitrification gene or (**B**) other nitrogen-cycling genes. Black bars above the graph indicate ODZ depths ($O_2 < 3$ µM).

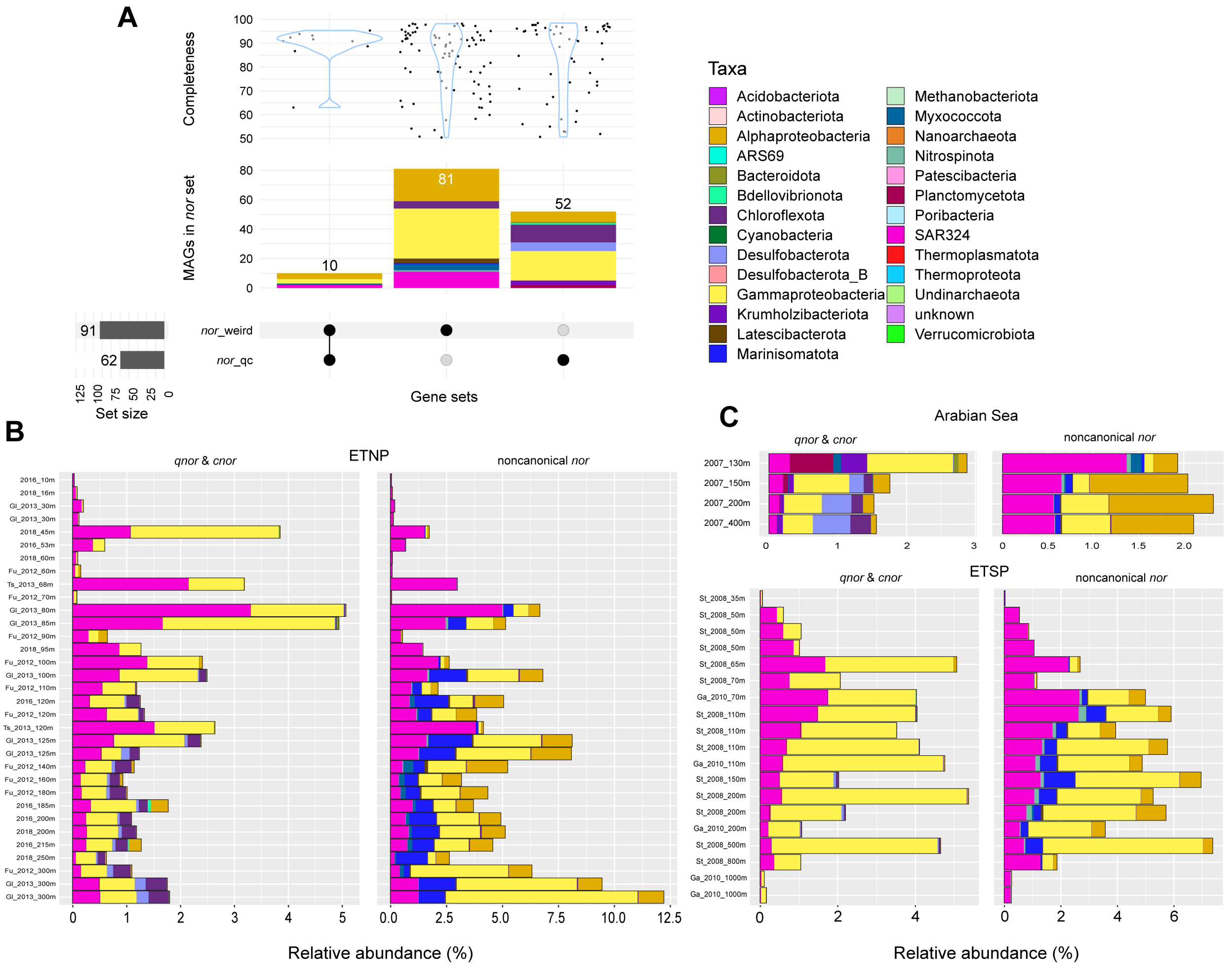

Supplementary Figure S8. A, The number of MAGs carrying canonical vs. non-canonical *nor* within the ODZ MAG collection. Top panel shows the number of MAGs colored by phylum-level taxonomy, with the exception of *Proteobacteria* which is colored by class. Bottom panel shows the genes within each gene set. Left bottom panel shows the number of MAGs carrying each type of *nor*. **B**, Relative abundances of MAGs across ETNP metagenomes carrying each type of *nor,* colored by phylum-level taxonomy, with the exception of *Proteobacteria* which is colored by class. **C**, Relative abundances of MAGs across ETSP and Arabian Sea metagenomes carrying each type of *nor,* colored by phylum-level taxonomy, with the exception of *Proteobacteria* which is colored by class. Top two panels correspond to Arabian Sea, while bottom two panels correspond to ETSP.

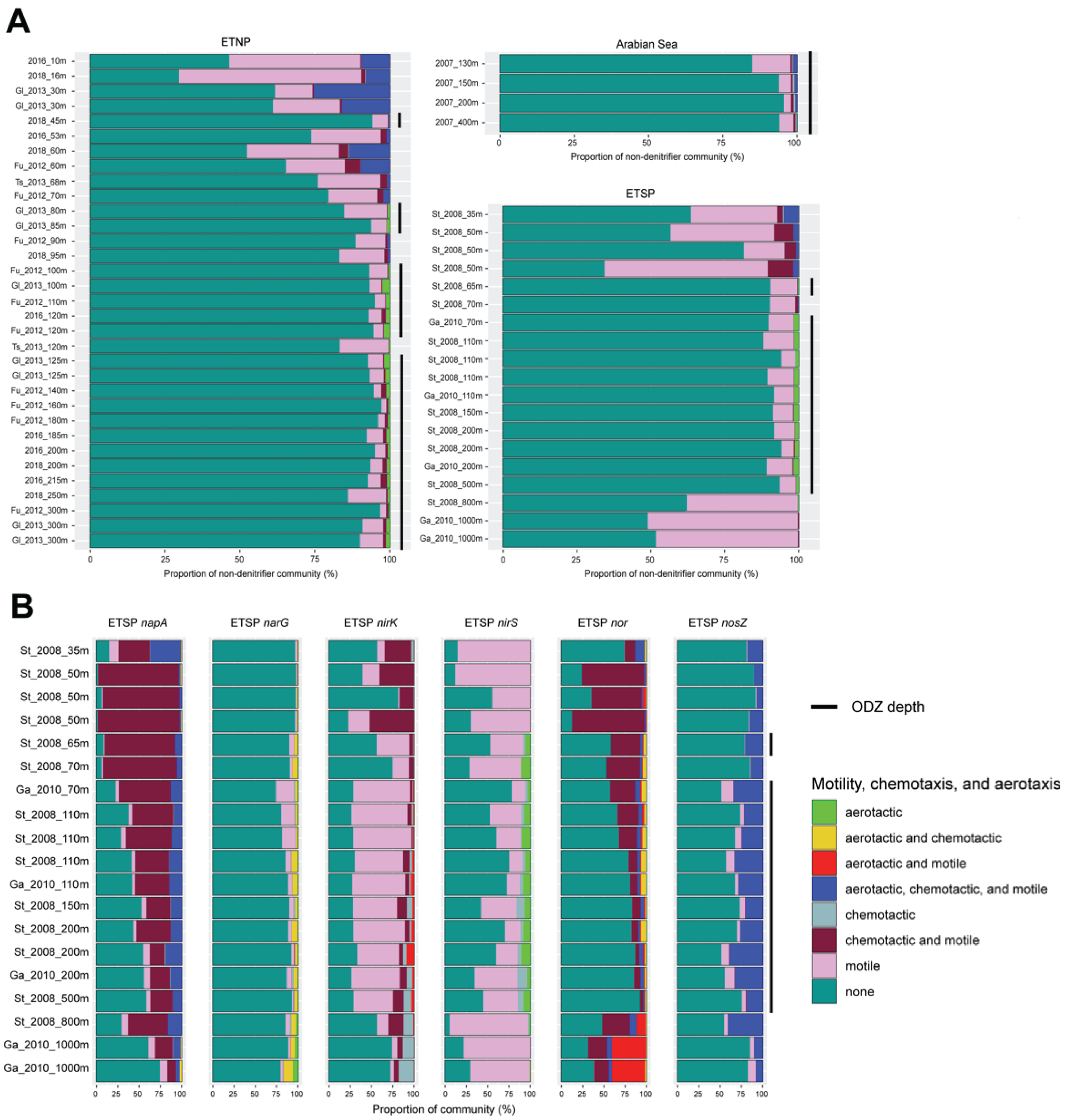

Supplementary Figure S9. (continued on next page)

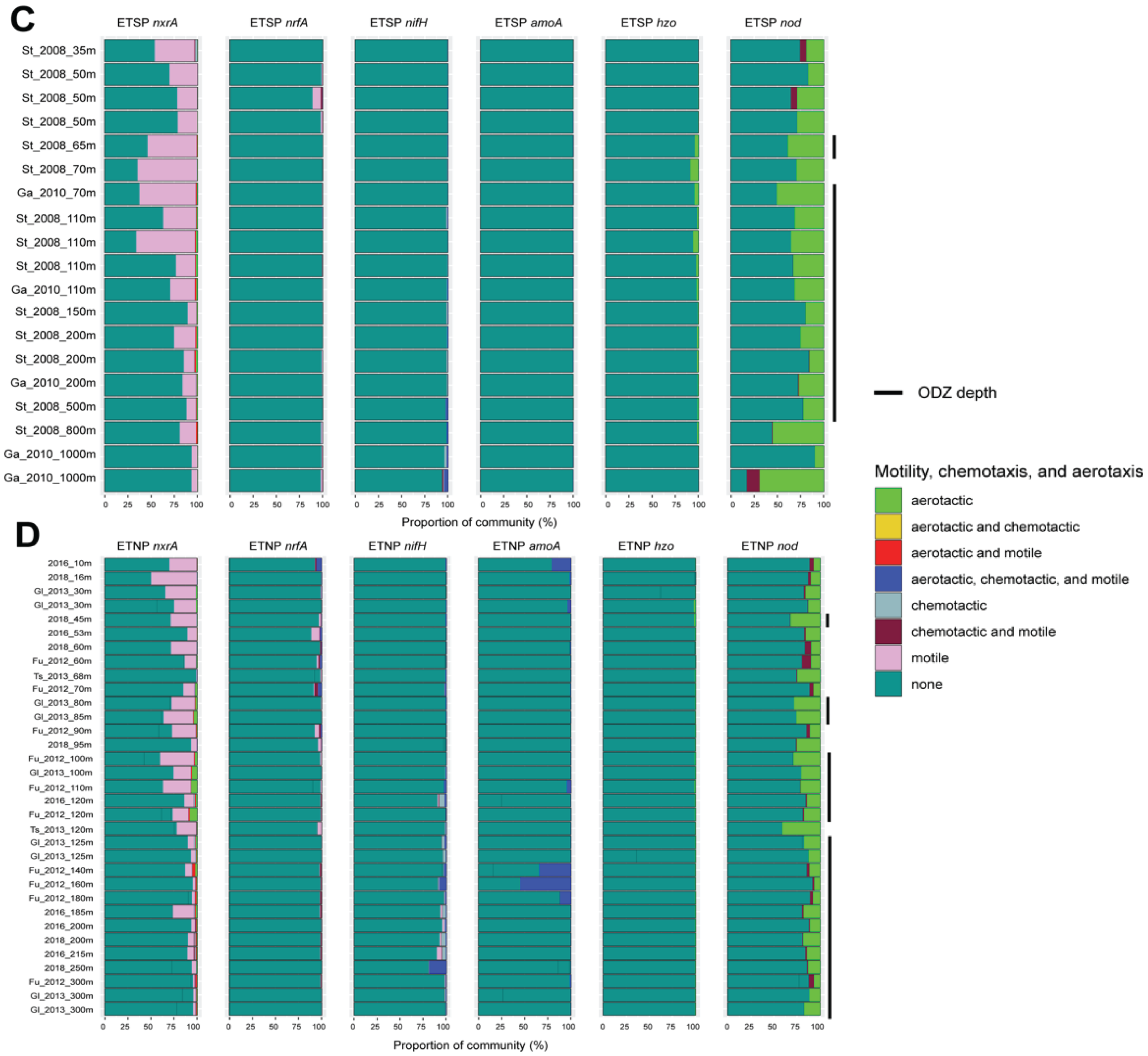

Supplementary Figure S9. A, Proportion of the non-denitrifying MAG community, scaled by relative abundance and colored by presence of motility, chemotaxis, and aerotaxis genes across all 3 ODZs. Graphs for the denitrifying community can be found in Figure 7A. **B**, Proportions of MAG communities carrying each queried denitrification gene, scaled by relative abundance and colored by presence of motility, chemotaxis, and aerotaxis, across all ETSP metagenomes. Graphs representing ETNP metagenomes can be found in Figure 7D. **C** and **D**, Proportions of the MAG communities carrying other nitrogen cycling genes, scaled by relative abundance and colored by presence of motility, chemotaxis, and aerotaxis for (**C**) the ETSP and (**D**) the ETNP. Proportions are calculated by (y / x) × 100%, where y = the relative abundances of all MAGs carrying the queried gene and falling into a specific motility, chemotaxis, or aerotaxis category and x = the relative abundances of all MAGs carrying the queried gene.

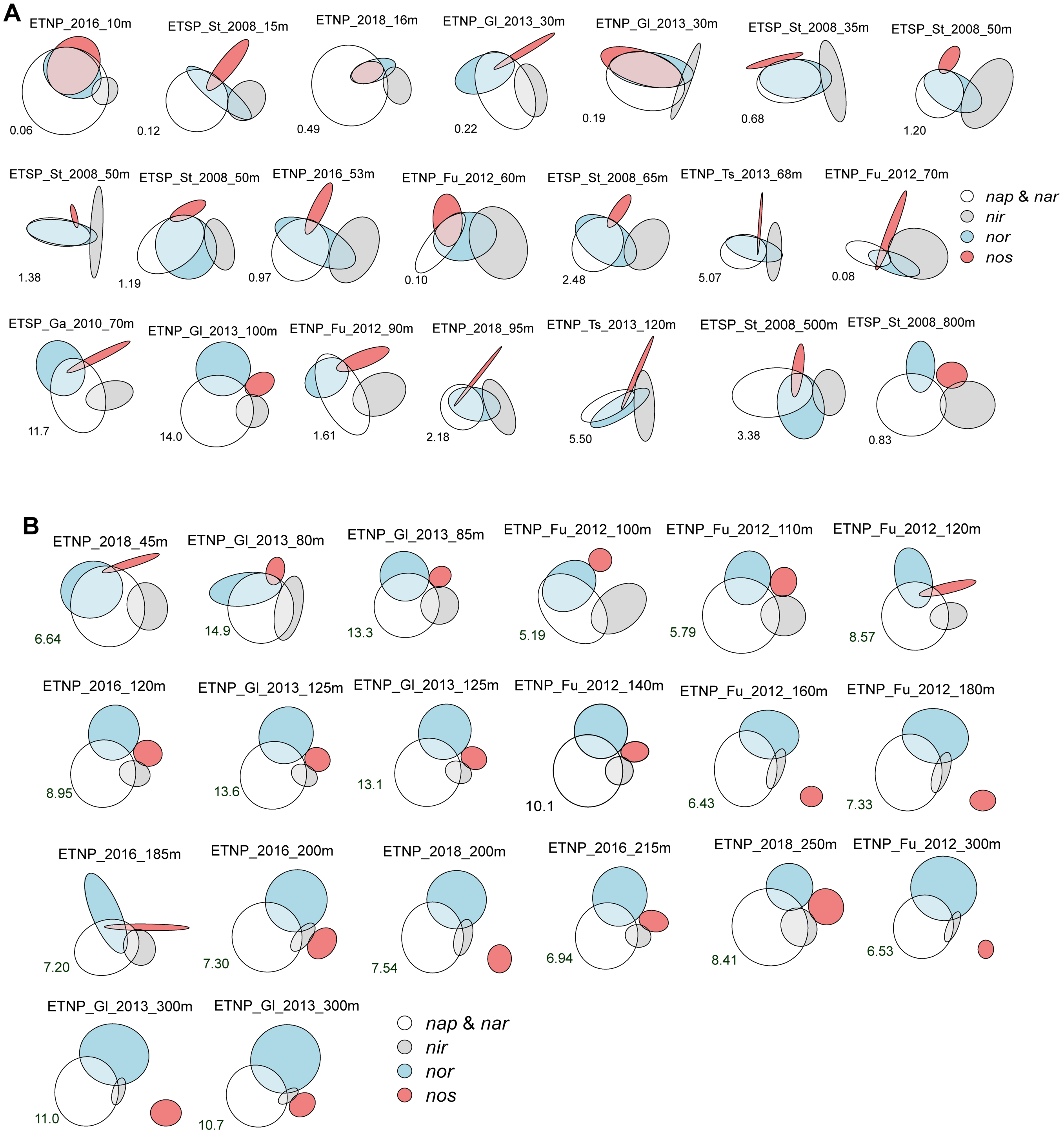

Supplementary Figure S10. (continued on next page)

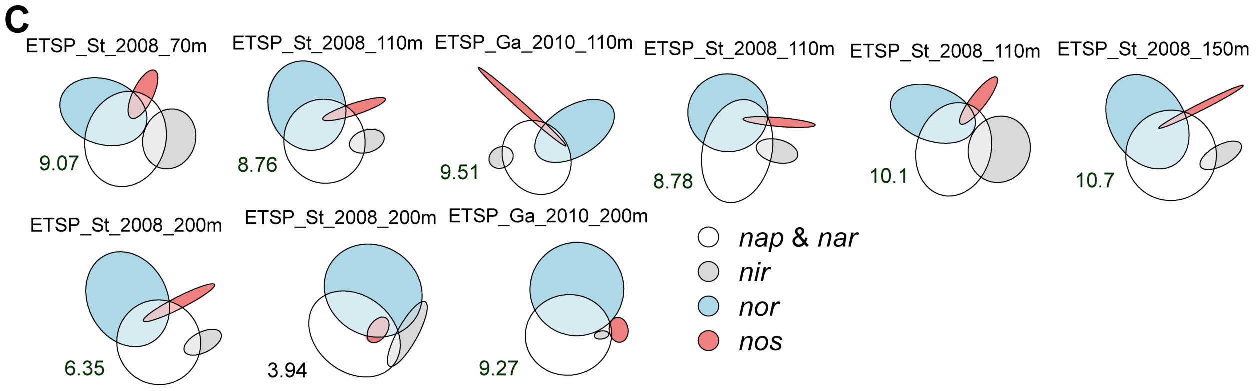

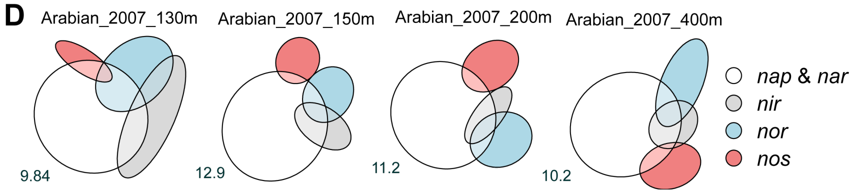

Supplementary Figure S10. Euler diagrams for (**A**) non-ODZ depths from the ETNP and ETSP, (**B**) ETNP ODZ depths, (**C**) ETSP ODZ depths, and (**D**) Arabian Sea ODZ depths. Circles and intersections are scaled to the total relative abundance of all MAGs possessing the genes for that step or step combination for each metagenome. The white circle corresponds to the relative abundance of MAGs with *napA, narG*, or both, and the numerical value for this relative abundance (%) is displayed on the left lower side of each diagram.

Supplementary Dataset S1. All ODZ metagenomes with associated nutrient and oxygen data, along with full CTD cast information for each sampling site corresponding to metagenomes.

Supplementary Dataset S2. All ODZ metagenomes included in this study, with associated information on assembly processes and quality information for assemblies.

Supplementary Dataset S3. All ODZ MAGs used in this study, with taxonomy, quality information, and gene presence/absence, and mapping results for all dereplicated ODZ MAGs against all metagenomes
